# Rod Monochromacy and the Coevolution of Cetacean Retinal Opsins

**DOI:** 10.1371/journal.pgen.1003432

**Published:** 2013-04-18

**Authors:** Robert W. Meredith, John Gatesy, Christopher A. Emerling, Vincent M. York, Mark S. Springer

**Affiliations:** 1 Department of Biology, University of California Riverside, Riverside, California, United States of America; 2 Department of Biology and Molecular Biology, Montclair State University, Montclair, New Jersey, United States of America; 3 Keck School of Medicine, University of Southern California, Los Angeles, California, United States of America; University of Michigan, United States of America

## Abstract

Cetaceans have a long history of commitment to a fully aquatic lifestyle that extends back to the Eocene. Extant species have evolved a spectacular array of adaptations in conjunction with their deployment into a diverse array of aquatic habitats. Sensory systems are among those that have experienced radical transformations in the evolutionary history of this clade. In the case of vision, previous studies have demonstrated important changes in the genes encoding rod opsin (RH1), short-wavelength sensitive opsin 1 (SWS1), and long-wavelength sensitive opsin (LWS) in selected cetaceans, but have not examined the full complement of opsin genes across the complete range of cetacean families. Here, we report protein-coding sequences for *RH1* and both color opsin genes (*SWS1*, *LWS*) from representatives of all extant cetacean families. We examine competing hypotheses pertaining to the timing of blue shifts in RH1 relative to *SWS1* inactivation in the early history of Cetacea, and we test the hypothesis that some cetaceans are rod monochomats. Molecular evolutionary analyses contradict the “coastal” hypothesis, wherein *SWS1* was pseudogenized in the common ancestor of Cetacea, and instead suggest that RH1 was blue-shifted in the common ancestor of Cetacea before *SWS1* was independently knocked out in baleen whales (Mysticeti) and in toothed whales (Odontoceti). Further, molecular evidence implies that *LWS* was inactivated convergently on at least five occasions in Cetacea: (1) Balaenidae (bowhead and right whales), (2) Balaenopteroidea (rorquals plus gray whale), (3) *Mesoplodon bidens* (Sowerby's beaked whale), (4) *Physeter macrocephalus* (giant sperm whale), and (5) *Kogia breviceps* (pygmy sperm whale). All of these cetaceans are known to dive to depths of at least 100 m where the underwater light field is dim and dominated by blue light. The knockout of both *SWS1* and *LWS* in multiple cetacean lineages renders these taxa rod monochromats, a condition previously unknown among mammalian species.

## Author Summary

The emergence of Cetacea (whales, dolphins, porpoises) represents a profound transition in the history of life. Living cetaceans have evolved a spectacular array of adaptations in association with their return to aquatic habitats. Aquatic environments impose challenging constraints on sensory systems, including vision, and the cetacean eye exhibits both anatomical and molecular specializations that enhance underwater sight. Most mammals have one photopigment (RH1) for dim-light vision and two photopigments (long wavelength-sensitive opsin [LWS], short wavelength-sensitive opsin [SWS1]) for daytime, color vision. By contrast, cetaceans have an inactivated copy of the gene that encodes SWS1. Here, we show that LWS is also inactivated in several cetacean lineages including the giant sperm whale, Sowerby's beaked whale, and balaenopteroids (rorquals plus gray whale). These cetaceans dive to depths of at least 100 meters where the underwater light field is dominated by dim, blue light. The knockout of both cone pigments renders these taxa rod monochromats, a condition that is previously unknown among mammalian species. Rod opsin remains functional in these taxa and is blue-shifted to increase its sensitivity to the available blue light that occurs in deep water conditions. These results further elucidate the molecular blueprint of modern cetacean species.

## Introduction

Cetacea [dolphins, porpoises, and whales] represents a remarkable example of aquatic specialization within Mammalia [1]. With their return to river and marine environments, the ancestors of modern toothed cetaceans (Odontoceti) and baleen whales (Mysticeti) underwent extensive modifications that included the evolution of novel structures [e.g., baleen plates, tail flukes], major anatomical rearrangements (e.g., telescoping of the skull, development of fore-flippers), the loss or reduction of typical mammalian traits (e.g., olfactory structures, hair, hindlimbs), and associated behavioral changes (echolocation, filter-feeding, deep-diving) [2]–[4]. At the genetic level this restructuring includes evidence of positive selection in loci related to high-frequency audition [5]–[7], brain size [8], [9], and flipper development [10], as well as degradation of genes related to olfaction [11]–[13], taste [14], tooth enamel formation [15]–[17], and vomeronasal chemoreception [18].

In the case of vision, aquatic environments impose challenging constraints, and the cetacean eye exhibits both morphological and molecular specializations that enhance underwater sight [19]. Possible morphological adaptations include an extensive reflective tapetum lucidum, a spherical lens with high refractive power, a relatively large cornea, and a rod-dominated retina, all of which enhance visual capabilities under dim light conditions [20], [21]. At the molecular level, most mammals have dichromatic color vision based on presence of three visual pigments, each of which is a G protein-coupled receptor that consists of an opsin protein moiety linked via a Schiff base to a retinal chromophore [22]. The three opsins that characterize most mammals include a rod opsin (RH1) and two cone opsins, short wavelength-sensitive opsin (SWS1) and long wavelength-sensitive opsin (LWS). Rods mainly function in dim light conditions (scotopic/night vision) whereas cones require more light (photopic vision) and are necessary with color vision. By contrast with most other mammals, all cetaceans that have been investigated are thought to be L-cone monochromats that possess an inactivated copy of SWS1 and two functional opsins, *RH1* and *LWS*, which are expressed in rod and L-cone cells of the retina, respectively [19], [23], [24].

Griebel and Peichl [19] and Peichl [24] suggested that retinal S-cones, which express SWS1 and are sensitive to blue wavelengths, were lost during an early, coastal period of cetacean evolution. Near-shore waters commonly have an underwater light spectrum that is red shifted owing to the absorption of blue light by organic and inorganic debris, and the loss of ‘jobless’ S-cones may have constituted an economical advantage in this environment by simplifying retinal and cortical visual information processing [19]. There are no inactivating frameshift mutations in *SWS1* that are shared by all odontocetes and mysticetes [23], but Griebel and Peichl [19] suggested that an unidentified genetic change, possibly in the promoter region, thwarted expression of the SWS1 protein in the common ancestor of crown Cetacea. Following the knockout of *SWS1*, crown cetacean lineages that independently conquered the open ocean were forced to shift λ_max_ [the wavelength of maximal absorption] of RH1 and LWS to bluer wavelengths because *SWS1* had previously been inactivated [19], [24]. By contrast, Bischoff et al. [25] offered an alternative scenario in which RH1 was blue shifted in the common ancestor of Cetacea. Specifically, Bischoff et al. [25] speculated that the ancestral cetacean RH1 possessed ^83^Asn, ^292^Ser, and ^299^Ala at three key tuning sites, as in the deep-diving giant sperm whale [*Physeter macrocephalus*], but stopped short of using explicit methods to reconstruct the ancestral RH1 sequence of Cetacea. If RH1 was blue-shifted in the common ancestor of Cetacea, then *SWS1* may have been inactivated independently in mysticetes and odontocetes, perhaps due to the inefficiency at S-cones at photon capture in dim light conditions [22].

Another intriguing hypothesis posits rod monochromacy, as opposed to L-cone monochromacy, in at least some cetaceans. McFarland [20] suggested that some cetaceans are probably rod monochromats in which S-cones, L-cones, and their associated opsin genes [*SWS1* and *LWS*, respectively] are lacking, so that vision is based entirely on rods and the rod opsin gene *RH1*. Immunocytochemical studies have failed to support this hypothesis and instead demonstrated the presence of L-cones in representative odontocete species belonging to the families Delphinidae and Phocoenidae [26]. More recently, Fasick et al. [21] reported the first partial L-cone opsin sequence (*LWS*) of a mysticete, *Eubalaena glacialis* (Atlantic right whale), and suggested that the L-cone opsin in this taxon is blue shifted, as are the L-cone opsins of representative odontocetes [27]. A potential shortcoming of this study is that Fasick et al. [21] only sequenced exons 3 and 5 of the *E. glacialis LWS* gene. In addition, *LWS* sequences have not been characterized from several other cetacean families including the deep-diving Ziphiidae, Physeteridae, and Kogiidae. Thus, McFarland's [20] suggestion that some cetaceans are rod monochromats remains to be tested by a more complete sampling of opsin gene sequences from a broader array of species.

Here, we report complete or nearly complete protein-coding sequences for all three opsin genes (*RH1*, *SWS1*, *LWS*) from representatives of all extant families of Cetacea and the cetacean sister group, Hippopotamidae. Previous studies have characterized the evolutionary patterns of individual cetacean opsins in isolation, but have not yet integrated information from all three retinal opsin genes (*RH1*, *LWS*, *SWS1*) into a single, comprehensive analysis. We utilized selection intensity estimates, ancestral sequence reconstructions, shifts in spectral tuning, and shared missense/frameshift mutations to infer the complex history of opsin evolution in Cetacea. Our reconstructions suggest that RH1 was blue-shifted in the common ancestor of Cetacea prior to the independent inactivation of *SWS1* on the stem mysticete and odontocete branches. *LWS*, in turn, was pseudogenized convergently in five different cetacean lineages [right whale plus bowhead, rorquals plus gray whale, Sowerby's beaked whale, giant sperm whale, pygmy sperm whale], all of which are deep divers that feed on bioluminescent organisms. The tandem inactivation of *SWS1* and *LWS* in these taxa presumably renders them rod monochromats, a condition that was previously unknown within Mammalia.

## Results

### Phylogenetic Analyses

**Figure 1:**
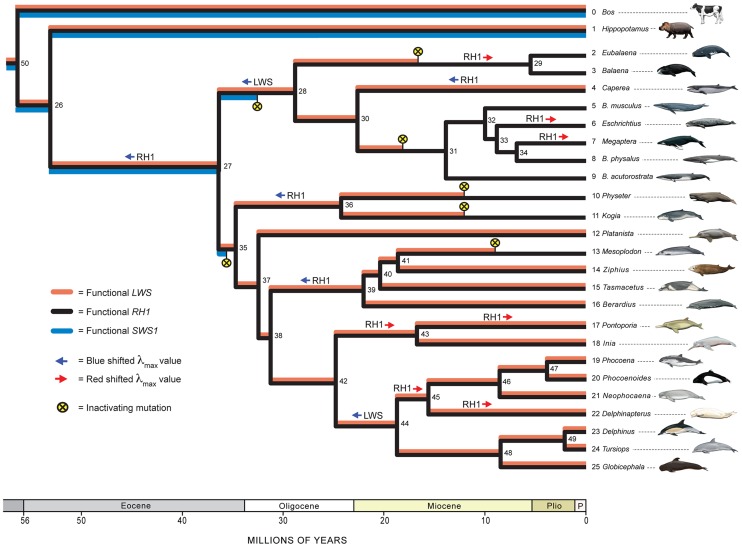
A hypothesis for the evolution of *LWS, SWS1*, and *RH1* in Cetacea and outgroups. Phylogenetic relationships and divergence times for Cetacea follow McGowen et al. [42] and for outgroups are as in Meredith et al. [101]. Nodes in the tree are numbered (1–50) and are referenced to in the main text. The coloration of branches indicates inferred functional *SWS1* (blue), functional *LWS* (red), and functional *RH1* (black); for example, all three opsins are reconstructed as functional in the common ancestor of Cetacea (node 26 to node 27), but only RH1 is functional in balaenopteroid baleen whales (node 31 and its descendants). Inferred inactivation events (frameshifts, premature stop codons, etc.) are marked by yellow circles with Xs, and are arbitrarily placed at the midpoints of branches to which these events were optimized. Inferred red and blue shifts in λ_max_ for LWS and RH1 are indicated by arrows at internodes. Geological time scale is shown at the bottom of the figure with time in millions of years (Plio = Pliocene; P = Pleistocene). For taxon names, the mysticete genus *Balaenoptera* is abbreviated as “*B.*”. Paintings are by Carl Buell.

Maximum likelihood trees based on *SWS1* exons plus introns, *SWS1* exons, *RH1* exons, and *LWS* exons are shown in Figures S1, S2, S3, S4. With a few exceptions, clades with high bootstrap support percentages (>90) on individual gene trees are in agreement with the species tree in Figure 1. All of the gene trees recovered Cetancodonta [Cetacea + Hippopotamidae], Cetacea, Mysticeti, Balaenidae, Balaenopteroidea, Physeteroidea, Ziphiidae, Iniidae + Pontoporiidae, Phocoenidae, Delphinidae, Delphinoidea, and Iniidae + Pontoporiidae + Delphinoidea [Delphinida]. Odontoceti was only recovered in the *SWS1* analyses, but conflicting nodes in the *RH1* and *LWS* trees had low bootstrap support values (≤53%).

### SWS1 Evolution

Inactivating mutations (frameshift indels, premature stop codons, disrupted intron splice sites, amino acid replacement at the Schiff's base counterion site) were apparent for all cetacean species in the *SWS1* alignment (Table 1), but were lacking in *SWS1* from the semiaquatic outgroup species, *Hippopotamus amphibius* (Figure 1). Although the *SWS1* genes of all cetacean species show evidence of mutational decay, no inactivating mutations map to the last common ancestral branch of Cetacea (Figure 1, node 26 to node 27). Instead, different molecular lesions define various sublineages of Cetacea (Table 1). An amino acid replacement (E113G; bovine RH1 numbering) at the Schiff's base counterion site that is thought to disrupt opsin-chromophore binding [23] optimizes to the stem branch of Odontoceti (Figure 1; node 27 to node 35), and a four base-pair frameshift deletion was derived on the stem branch of Mysticeti (Figure 1; node 27 to node 28). These independent inactivating mutations imply that *SWS1* was pseudogenized convergently in the two major subclades of Cetacea (Figure 1, Figure S5).

**Table 1:** Summary of inactivating mutations in cetacean *SWS1* and *LWS* genes.

SWS1 Mutations			
Taxon	Number of Exon or Intron	Inactivating Mutation Including Alignment Number and Nucleotide Position(s)	Location on Tree (Ancestral Node Number: Descendant Node Number)
*Tursiops truncatus*	Exon 1	1-bp frameshift deletion (Alignment1:34)	49∶24
Mysticeti, *Mesoplodon bidens*, *Platanista minor*, *Phocoenoides dalli* (polymorphic), *Delphinapterus leucas* (polymorphic)	Exon 1	4-bp frameshift deletion (Alignment1:270–273)	27∶28, 41∶13, 37∶12, 47∶20, 45∶22
*Physeter macrocephalus*	Exon 1	1-bp frameshift insertion (Alignment1:310)	36∶10
*Globicephala melas*	Exon 1	50-bp frameshift deletion (Alignment1:261–311)	48∶25
*Pontoporia blainvillei*	Exon 1	2-bp frameshift deletion (Alignment1:308–309)	43∶17
Odontoceti	Exon 1	E113G amino acid replacement at the Schiff's base counterion site (bovine rhodopsin numbering) (Alignment1:338–340)	27∶35
*Berardius bairdii*	Intron 1	GT to GC splice site mutation (Alignment1:363–364)	39∶16
*Eschrichtius robustus*	Exon 2	8-bp frameshift deletion (Alignment1:808–815)	33∶6
*Globicephala melas*	Exon 3	4-bp frameshift insertion (Alignment1:1438–1441)	48∶25
*Caperea marginata*	Intron 3	GT to GC splice site mutation (Alignment1:1489–1490)	30∶4
*Platanista minor*	Exon 4	1-bp frameshift insertion (Alignment1:2201)	37∶12
*Kogia breviceps*	Exon 4	1-bp frameshift deletion (Alignment1:2336)	36∶11
*Pontoporia blainvillei*	Exon 4	2-bp frameshift insertion (Alignment1:2337–2338)	43∶17
Physeteroidea	Intron 4	GT to GA splice site mutation (Alignment1:2382–2383)	35∶36
*Tursiops truncatus*	Exon 5	1-bp deletion (Alignment1:4079)	49∶24
*Balaenoptera acutorostrata*	Exon 5	2-bp frameshift deletion (Alignment1:4103–4104)	31∶9

LWS Mutations			
Taxon	Number of Exon or Intron	Inactivating Mutation Including Alignment Number and Nucleotide Position(s)	Location on Tree (Ancestral Node Number: Descendant Node Number)
Balaenopteroidea	Exon 1	22 bp deletion that includes first two bp of initiation codon in exon 1 (Alignment4:99–120)	30∶31
*Kogia breviceps*	Exon 1	44-bp frameshift deletion (Alignment4:120–163)	36∶11
*Balaenoptera musculus*	Exon 1	1-bp frameshift deletion (Alignment4:169)	32∶5
Balaenopteroidea	Exon 2	1-bp frameshift deletion (Alignment5:238)	30∶31
*Mesoplodon bidens*	Exon 2	4-bp frameshift insertion (Alignment5:409–412)	41∶13
*Kogia breviceps*	Intron 2	GT to CT splice site mutation (Alignment5:504–505)	36∶11
*Eubalaena australis*	Intron 2	AG to CG splice site mutation (Alignment6:5–6)	29∶2
*Physeter macrocephalus*	Exon 5	28-bp frameshift deletion (Alignment8:186–213)	36∶10
*Physeter macrocephalus*	Exon 5	1-bp frameshift deletion (Alignment8:251)	36∶10
Balaenidae	Intron 5	AG to GG splice site mutation (Alignment9:32–33)	28∶29
*Eubalaena australis*	Exon 6	7-bp frameshift deletion (Alignment9:47–53)	29∶2

Inactivating mutations include frameshifts, Schiff's base counterion site mutations, and splice site mutations. All alignments are provided in Text S3. Inactivating mutations are also cross-referenced to branches in Figure 1.

Estimates of ω (dN/dS) on different branches of the cetacean tree are consistent with parallel knockouts of *SWS1* in Odontoceti and in Mysticeti. The ω estimate for *SWS1* on the stem Cetacea branch (Figure 1, node 26 to node 27), just prior to the two inferred inactivating mutations, suggests a pattern of purifying selection based on analyses with two different codon frequency models (ω = 0.31, 0.35). Likewise, a signature of strong purifying selection (ω = 0.16, 0.17) was inferred on the stem Odontoceti branch (node 27 to node 35) (Table 2). Neutrality is predicted on the stem odontocete branch if *SWS1* had previously been inactivated on the stem cetacean branch [19], but statistical tests rejected this hypothesis (Table 2). The ω estimate (0.75, 0.83) for the stem mysticete branch (node 27 to node 28), in turn, is only slightly lower than expected for complete neutrality (ω = 1.0) and suggests that pseudogenization occurred very early on this branch. Estimates of ω for crown odontocete + crown mysticete branches are in agreement with expectations for neutrality (Table 2), and in conjunction with numerous frameshift indels within these clades (Table 1) imply a release from selective constraints after the occurrence of inactivating mutations on the stem odontocete and stem mysticete branches (Figure 1).

**Table 2:** Results of χ^2^-tests for nonsynonymous and synonymous substitutions in *SWS1*.

CF = 3 (774.4 non-synonymous sites: 239.6 synonymous sites)							
	Observed		Expected				
Branch(es)	N	S	N	S	ω	χ^2^	*p* value
1. Crown odontocete + crown mysticete branches	193.3	47.8	184.2	56.9	1.25	1.91	0.17
2. Odontocete stem branch	2.1	4	4.66	1.44	0.16	5.95	0.015*
3. Mysticete stem branch	6.2	2.3	6.49	2.01	0.83	0.06	0.81

CF = 2 (765.4 non-synonymous sites: 248.6 synonymous sites)							
	Observed		Expected				
Branch(es)	N	S	N	S	ω	χ^2^	*p* value
1. Crown odontocete + crown mysticete branches	195.2	48.9	184.3	59.8	1.29	2.63	0.10
2. Odontocete stem branch	2.1	4	4.60	1.50	0.17	5.56	0.018*
3. Mysticete stem branch	6.3	2.7	6.79	2.21	0.75	0.15	0.70

χ^2^-tests compared the observed versus expected numbers of nonsynonymous and synonymous substitutions for *SWS1* based on a neutral model of evolution (ω = 1). All three different branch categories are expected to exhibit neutral ω values according to the “coastal” hypothesis of *SWS1* evolution. CF = codon frequency model; N = nonsynonymous; S = synonymous; ω = dN/dS;

* = significant *p* values.

### RH1 Evolution

No inactivating mutations (frameshift indels, splice site mutations) were apparent in the *RH1* alignment, implying that RH1 is functional in all of the species that were surveyed (Figure 1).

Ancestral amino acid sequences at key tuning sites (83, 292, 299) in Cetacea [21], [25] are shown in Table 3 for internal nodes with inferred blue or red shifts in λ_max_. Amino acid changes from DAS to NSS on the stem cetacean branch [node 26 to node 27] resulted in an inferred blue shift from 501 to 484 nm. Additional blue shifts (484 to 479 nm) are inferred in *Caperea*, in stem Physeteroidea (node 35 to node 36), and in stem Ziphiidae (node 38 to node 39) based on an amino acid changes at site 299 (NSS to NSA) that occurred independently in these three lineages. Seven red shifts were reconstructed in Cetacea, including three in Mysticeti (stem Balaenidae [node 28 to node 29], *Megaptera*, *Eschrichtius*) and four in Odontoceti (stem Iniidae + Pontoporiidae [node 42 to node 43], *Pontoporia*, stem Monodontoidea [node 44 to node 45], *Delphinapterus*) (Table 3).

**Table 3:** Amino acids at key tuning sites for RH1 [21], [25] and LWS [36].

Branch Node Numbers (Ancestor to Descendant)	Branch Name	RH1		LWS	
		Amino Acid Changes at Key Tuning Sites(83 292 299)	λ_max_ (nm) Change on Branch	Amino Acid Changes at Key Tuning Sites(180 197 277 285 308)	λ_max_ (nm) Change on Branch
26 to 27	Stem Cetacea	DAS to NSS	501 to 484	No changes	No change
27 to 28	Stem Mysticeti	No change	No change	AHYTA to AHYTS	552 to 522–531
28 to 29	Stem Balaenidae	NSS to NAS	484 to 493	No change	No change
30 to 4	*Caperea*	NSS to NSA	484 to 479	No change	No change
33 to 6	*Eschrichtius*	NSS to NAS	484 to 493	No change	No change (pseudogene)
34 to 7	*Megaptera*	NSS to DSS	484 to 492	No change	No change (pseudogene)
35 to 36	Stem Physeteroidea	NSS to NSA	484 to 479	No change	No change
38 to 39	Stem Ziphiidae	NSS to NSA	484 to 479	No change	No change
42 to 43	Stem Iniidae + Pontoporiidae	NSS to NAS	484 to 493	No change	No change
43 to 17	*Pontoporia*	NAS to DAS	493 to 501	No change	No change
43 to 18	*Inia*	NAS to NAT*	493 to ?	AHYTA to AHYTP*	552 to ?
44 to 45	Stem Monodontidae + Phocoenidae	NSS to DSS	484 to 492	No change	No change
45 to 22	*Delphinapterus*	DSS to DAS	492 to 501	No change	No change
42 to 44	Stem Delphinoidea	No change	No change	AHYTA to AHYTS	552 to 522–531

Node numbers correspond to Figure 1. RH1 λ_max_ values are based on Fasick et al. [21] for DAS and Bischoff et al. [25] for all other amino acid combinations. LWS λ_max_ values are based on Fasick et al. [21].

* = λ_max_ value unknown.

Among ten other amino acid sites that have been linked to spectral tuning in vertebrates [28], eight (sites 96, 102, 122, 183, 253, 261, 289, 317) are invariant among the cetaceans and the hippopotamid that were included in our taxon sampling, site 194 exhibits four amino acid replacements within Cetacea, and site 195 shows an amino acid replacement [L to P] on the stem Cetacea branch and four replacements within Cetacea.

Analyses with Codeml rejected site models 2 and 8, which add an extra category for positively selected sites, in favor of models 1 and 8a, respectively. By contrast, branch-site analyses with two different codon frequency models (CF) provided statistically significant support for a bin of five positively selected sites (7, 83, 123, 266, 292) on branches with λ_max_ changes (CF2: P = 0.00036, ω = 5.53; CF3: P = 0.00018, ω = 6.43). Three of the five positively selected sites (83, 266, 292) have probabilities >0.95 of membership in this bin.

### LWS Evolution

Inactivating mutations are apparent in *LWS* sequences from ten cetacean species (Figure 1, Figure S6, Table 1). Reconstructions of ancestral sequences imply eight frameshift indels and three splice site disruptions within Cetacea, with convergent inactivation of *LWS* on the following five branches (Figure 1): *Physeter macrocephalus*, *Kogia breviceps*, *Mesoplodon bidens*, stem Balaenidae (node 28 to node 29), and stem Balaenopteroidea (node 30 to node 31). All of these separate knockouts of *LWS* postdate prior inactivations of *SWS1* and therefore result in rod monochromacy (Figure 1).

Estimates of ω throughout the species tree generally are consistent with multiple, independent knockouts of *LWS* within Cetacea. Branches reconstructed as functional for *LWS* exhibit a strong signature of purifying selection (ω = 0.09, 0.10). By contrast, ω estimates on “transitional” branches [16], where inactivating mutations in *LWS* were reconstructed, generally show elevated rates of nonsynonymous substitution (*Physeter*: ω = 0.38, 0.41, *Kogia*: ω = 0.73, 0.78, stem balaenopteroid branch: ω = 0.34, 0.37). Exceptions are the short transitional branches for stem Balaenidae (1.5 to 1.7 inferred substitutions, ω = 0.0001) and *Mesoplodon* (2.4 to 3.1 inferred substitutions, ω = 0.13, 0.19). Branches within crown Balaenopteroidea (node 31 and descendant branches) plus crown Balaenidae (node 29 and descendant branches), which are interpreted as pseudogenic based on the prior occurrence of inactivating mutations, have an ω value based on two codon models (0.69, 0.70) that does not deviate significantly from neutral expectations (ω = 1.00) based on χ^2^-tests.

Reconstructions of ancestral amino acid sequences at five key tuning sites (amino acids 180, 197, 277, 285, 308) [21], [25] are shown in Table 3 for branches with inferred shifts in λ_max_. For the five tuning sites, AHYTA (λ_max_ = 552 nm) is the inferred ancestral condition for Cetancodonta (node 26) and for the last common ancestor of extant cetaceans (node 27). Three changes at LWS tuning sites were reconstructed within Cetacea. Parallel changes from AHYTA to AHYTS on the stem Mysticeti branch [node 27 to node 28] and on the stem Delphinoidea branch (node 42 to node 44) imply blue shifts from 552 nm to 522–531 nm. A change from AHYTA to AHYTP was reconstructed on the terminal *Inia* branch, but the functional effect of A308P is unknown (Table 3).

Analyses with Codeml rejected site models 2 and 8, which add an extra category for positively selected sites, in favor of models 1 and 8a, respectively. Similarly, positively selected sites were not identified in branch-site analyses.

## Discussion

### Opsin Evolution in Cetacea

Here, we assembled complete or nearly complete protein-coding sequences for *RH1*, *SWS1*, and *LWS* for representatives of all extant cetacean families. These sequences, in combination with molecular evolutionary analyses, permit a detailed, synthetic reconstruction of opsin evolution in Cetacea (Figure 1).

Recent phylogenetic hypotheses imply that the aquatic ancestry of Cetacea extends back to its last common ancestor with the semi-aquatic Hippopotamidae in the early Eocene, >50 Ma [4], [29], [30]. Whales and hippos share a variety of “aquatic” specializations including sparse hair, loss of sebaceous glands, and the ability to birth and nurse underwater [4], [31], [32], but these features traditionally have been interpreted as parallel evolutionary derivations in these two lineages. Given the hypothesis that the common ancestor of cetaceans and hippos was aquatic/semi-aquatic (Figure 1, node 50 to node 26), shared mutations in opsin genes that enhance vision in aquatic environments might be expected in whales and hippos. ML reconstructions of ancestral opsin sequences imply only two replacement substitutions (LWS: E41D; RH1: L216M) on the stem lineage. These replacements are not at key tuning sites and fail to provide compelling evidence for an aquatic shift in opsin properties in the common ancestor of hippos and whales.

Following divergence from Hippopotamidae, the unique evolutionary history of Cetacea began on the stem cetacean branch (Figure 1, node 26 to node 27). The fossil record indicates that the stem cetacean lineage was marked by a profound transition in anatomy from primitive semi-aquatic forms to obligately aquatic taxa with vestigial hindlimbs [3], [33]–[35]. Ancestral reconstructions imply that stem cetaceans retained dichromatic color vision with functional *SWS1*, *LWS*, and *RH1* as in *Hippopotamus* and more distantly related artiodactyls; a blue shift in RH1 also occurred on the stem cetacean branch (Figure 1). Specifically, the amino acid array at three key tuning sites (83, 292, 299) [21], [25] changed from DAS to NSS, with an inferred λ_max_ shift from 501 to 484 nm. Our ML reconstruction supports Bischoff et al.'s [25] hypothesis that RH1 was blue shifted on the stem cetacean branch, but contradicts their assertion that the ancestral cetacean expressed the amino acids NSA as in deep-diving physeteroids.

In addition to replacements at sites 83 and 292, a change at tuning site 195 (K to T) of RH1 occurred on the stem cetacean branch. This change from a polar amino acid to a positively charged residue has been retained in the deep-diving physeteroids (giant sperm whale, pygmy sperm whale). The inferred shift in λ_max_ that results from a K to T replacement at this site, if any, remains to be investigated with mutagenesis studies. Unlike tuning sites 83, 292, and 299, that are situated in transmembrane regions of RH1 and are in close proximity to the chromophore, site 195 is positioned in the luminal face of RH1 [36]. The nature of long distance interactions between this amino acid site and the chromophore are unknown [36].

The basal split in Cetacea defines the separation of Odontoceti from Mysticeti, and also marks the evolution of profound changes in anatomy/feeding strategy in both clades [4], [37]. Echolocation capabilities and degradation of olfactory structures were derived on the stem odontocete branch (Figure 1, node 27 to node 35), whereas the transition to bulk filter feeding with a keratinous baleen sieve evolved on the stem mysticete branch (Figure 1, node 27 to node 28). These divergent specializations represent changes in feeding style that would be expected to impact demands on visual systems.

Following the blue shift in RH1 on the stem cetacean branch, *SWS1* was inactivated independently in stem odontocetes and in stem mysticetes, coincident with the evolution of divergent specializations in these two clades (Figure 1). Two lines of evidence support this reconstruction and argue against an earlier knockout of *SWS1* in the common ancestor of Cetacea. First, comprehensive sequencing of *SWS1* exons and introns revealed no shared inactivating mutations common to all extant cetaceans. Odontocetes have a common missense mutation at the Schiff's base counterion site (E113G) that disrupts opsin-chromophore binding [23]. Mysticetes, in turn, share a 4-bp frameshift mutation in exon 1 of *SWS1* that results in a premature stop codon. Frameshift indels in the same position occur in several odontocetes, but these deletions are most parsimoniously reconstructed as convergent between Mysticeti and multiple odontocete subclades (Text S1). Several mutations that disrupt intron boundaries were identified, but in all cases these substitutions map to branches within Odontoceti or within Mysticeti. Second, dN/dS values on the stem odontocete and stem mysticete branches should indicate an absence of selective constraints if *SWS1* was inactivated earlier in the common ancestor of Cetacea. Estimates of dN/dS (0.75, 0.83) for the stem mysticete branch are consistent with neutral evolution (dN/dS = 1.00), but neutrality was rejected given the low dN/dS estimates (0.16, 0.17) for the stem odontocete branch, indicative of purifying selection and thus functionality after the split between Odontoceti and Mysticeti (Figure 1, node 27; Table 2).

Together, our reconstructions for the evolution of RH1 and *SWS1* contradict the coastal knockout hypothesis [19], [24]. This scenario postulates that *SWS1* was inactivated during an early amphibious phase of cetacean history when semi-aquatic whales occupied coastal waters that absorbed blue light, and that RH1 was subsequently blue shifted in crown cetaceans that moved to open ocean environments dominated by blue light. The coastal knockout hypothesis requires an as yet undiscovered inactivating mutation in *SWS1*, perhaps in the promoter region of this gene or at splice sites [23]. Instead, our results fit the hypothesis that RH1 was blue shifted in the common ancestor of Cetacea, and that *SWS1* was convergently knocked out in Odontoceti and in Mysticeti after cetaceans had invaded open ocean habitats. This is perhaps surprising given that SWS1 is well suited to detect the blue light that dominates the open ocean. However, the relative scarcity of S cones in the mammalian retina, which diminishes the efficiency of photon capture under dim light conditions, may have predisposed S-cones to eventual loss through relaxed selection [22]. By contrast, rods are much more efficient at photon capture under dim light conditions because of their higher density in the mammalian retina and their integration with large, sparsely distributed ganglion cells that sum photon detection over huge receptive fields [38]. The preferential retention of a functional copy of *LWS* rather than *SWS1* in some cetaceans may reflect the higher density of L-cones and their greater impact on visual acuity [22].

In addition to the blue shift of RH1 in the ancestral cetacean branch, several amino acid replacements in RH1 imply further adjustments in λ_max_ (Figure 1). These shifts are generally consistent with the photic environments that are occupied by different cetacean species [20], [25]. Among these changes are blue shifts in deep-diving physeteroids [sperm whales] and in ziphiids [beaked whales], a red shift in the common ancestor of *Inia* and *Pontoporia*, both of which are found in shallow water environments, and red shifts in several mysticetes [e.g., *Eschrichtius*, *Megaptera*]. Bischoff et al. [25] suggested that the red-shifted pigments that occur in some mysticetes are better adapted to relatively shallower foraging environments than the ancestral mysticete pigment. The blue shifts in Physeteroidea and Ziphiidae occured in parallel and in both cases involve amino acid replacements [serine to alanine] at tuning site 299 [21], [25]. Sperm whales and beaked whales rank among the deepest diving mammals and specialize on a cephalopod-rich diet [39]. Several phylogenetic studies of anatomical evidence grouped these suction feeding species, presumably based on convergent character states related to their deep diving habits [40], [41], but most recent work indicates that ziphiids are more closely related to dolphins and porpoises than to physeteroids [37], [42], [43].

Nozawa et al. [44] suggested that Yang's [45] codeml program is not useful for identifying adaptive sites in visual pigments. Our results support Nozawa et al.'s [44] finding that site analyses fail to identify adaptive changes in visual pigments. However, branch-site tests identified five codons in *RH1* that have evolved under positive selection on branches with inferred changes in λ_max_. The ω value for the five positively selected sites is well above one (5.53–6.43), and supports the hypothesis that changes affecting λ_max_ in cetacean RH1 proteins are adaptive. The failure of site analyses to detect positively selected sites in RH1 may be a consequence of mixing positive selection on foreground branches with purifying selection on background branches. Nozawa et al. [44] criticized branch-site tests [45]–[47] for their proclivity to generate false positive results based on simulations, but Yang et al. [48] correctly noted that false positives only occurred in 32/14,000 cases, which is much lower than the nominal significance level (5%) and demonstrates that the branch-site test is conservative. Among the positively selected sites, two (83, 292) are known tuning sites that in part are the basis for inferring changes in λ_max_ (Figure 1). Changes at site 83 may also be important in dim-light conditions because the amino acid at this position affects the rate at which photoreceptor cells generate electrical signals [49]. The other three sites (7, 123, 266) have not been predicted to affect λ_max_. Site 7 occurs in the extracellular domain, site 123 occurs in transmembrane helix III, and site 266 occurs in transmembrane helix 6 [50]. The functional consequences of mutations at these amino acid positions in cetacean RH1 sequences remain unknown, although conformational changes associated with transmembrane domains III and VI of G protein-coupled receptors may be important in receptor activation [51].

Changes in LWS spectral sensitivity coincide with deployment of cetaceans to diverse aquatic habitats (Figure 1). A blue shift in LWS in stem mysticetes co-occurs with an *SWS1* frameshift mutation on the same branch, although the sequence of these events is unclear. An additional LWS blue shift in λ_max_ maps to the common ancestor of Delphinoidea [dolphins, porpoises, beluga], but the most striking feature of *LWS* evolution in Cetacea is the convergent knockout of this gene in five different lineages: Balaenopteroidea (rorquals and gray whale), Balaenidae (bowhead and right whale), *Mesoplodon bidens* (Sowerby's beaked whale), *Physeter macrocephalus* (giant sperm whale), and *Kogia breviceps* (pygmy sperm whale) (Figure 1). Given that *SWS1* is also debilitated in each of these species (Figure 1), the genetic data imply that these taxa are rod monochromats. This iterated degeneration of cetacean *LWS* was not apparent in earlier studies because complete protein-coding *LWS* sequences had been generated for only a few cetacean species [21].

### Rod Monochromacy in Cetacea

Historically, the pure rod retina has been proposed as the “extreme” adaptation to low light levels [52]. Walls [52] and McFarland [20] suggested the possibility of rod monochromacy in at least some cetaceans. More generally, early studies on retinal anatomy hinted at this condition in a variety of nocturnal and aquatic mammalian species with rod dominated retinas, including night monkeys, lemurs, tarsiers, chinchillas, seals, and bats [52]–[57]. Recent work has shown that representative cetaceans are instead L-cone monochromats and retain a functional copy of *LWS* [21], [26]. Similarly, primates, rodents, pinnipeds, and bats that were previously hypothesized to be rod monochromats are now known to be L-cone monochromats with functional *LWS* or even cone dichromats with functional *LWS* and *SWS1* [22], [58]–[61]. The present survey of cetacean opsins, which documents pseudogenization of both *SWS1* and *LWS* in multiple cetacean lineages, vindicates McFarland's [20] hypothesis that some cetaceans are rod monochromats (Figure 1). To our knowledge these are the only known examples of rod monochromacy in Mammalia or even Amniota. The observation that five independent derivations of mammalian rod monochromacy are all clustered within Cetacea is striking, and suggests that one or more features of cetacean biology have been pivotal in driving the degenerative pattern of opsin evolution in this aquatic clade.

The naked mole rat (*Heterocephalus glaber*) is the only other mammal, aside from the cetacean species characterized here, that is known to lack a functional copy of *LWS*, but *H. glaber* retains an intact *SWS1* and is interpreted as a cone monochromat [62]. This condition contrasts with other mammalian cone monochromats [pinnipeds, dolphins, porpoises, some procyonids, some rodents, some bats] that combine a pseudogenic *SWS1* with a functional copy of *LWS* [22], [23], [28], [60], [63]–[71]. It has been suggested that cetacean cone monochromats [e.g. bottlenose dolphin, *Tursiops truncatus*] can distinguish colors, possibly via interactions between LWS and RH1 [38], [72], but any vestiges of color vision presumably have been lost in the various rod monochromatic cetacean species documented here (Figure 1).

Among other vertebrates, rod monochromatic taxa are rare and to our knowledge have only been documented in bony and cartilaginous fishes [73]–[81], caecilians [22], [82], and the cave salamander *Proteus anguinus* [83], although presumed rod monochromacy based entirely on immunocytochemistry, microscopy or spectral analysis does not preclude the possibility that other minor visual pigment classes exist [77], [81], [83]. Most of the rod monochromatic fish species inhabit the deep sea or are nocturnal; caecilians are generally fossorial and/or nocturnal with poorly developed eyes; and the cave salamander *Proteus* lives in a virtually light-free environment.

The phylogenetic evidence for multiple, independent knockouts of both *SWS1* and *LWS* within Cetacea raises the question of why convergent pseudogenization and rod monochromacy evolved in this clade but not in other mammalian groups. All rod monochromatic cetacean species that were genetically characterized in our survey are capable of diving to depths that exceed 100 m, with sperm and beaked whales ranking among the deepest diving mammals [39], [84]–[88]. The selective pressures on mammalian retinal opsins in deep-water habitats are drastically different from those on land. In the open marine environment, the electromagnetic radiation of visible light is weakened with depth due to absorption and scattering [89]. In the mesopelagic zone (150–1000 m), down-welling sunlight becomes more monochromatic and the spectrum shifts towards shorter, bluer wavelengths with depth [81], [82]. Below 1000 m (bathypelagic zone), there is no down-welling sunlight and localized bluish bioluminescence becomes the predominant source of light [90].

A rod-dominated retina is advantageous in dim light conditions [38]. Therefore, the convergent pseudogenization of *SWS1* and *LWS* in multiple cetacean lineages may be an adaptation to deep-water habitats and/or feeding at night on bioluminescent invertebrate prey. Cone opsins have a higher rate of thermal activation [i.e., dark noise] than RH1 [91] and may interfere with rod sensitivity under scotopic conditions. Thus, combined *SWS1* and *LWS* pseudogenization may have increased RH1 sensitivity in physeteroids and ziphiids that feed in the mesopelagic and bathypelagic zones. Echolocation is a key specialization that has enabled odontocete taxa such as these to forage at night and at great depths on individual prey items, in particular cephalopods [92]; rod monochromatism may be an additional adaptive feature that has enabled predation at depth. Balaenopteroid and balaenid mysticetes are not known to feed in the bathypelagic zone, do not echolocate, and instead batch filter aggregations of small prey items. However, baleen whales do feed at night and much of their diet is composed of bioluminescent prey including krill [93], [94]. The ability to take advantage of this huge resource offers a compelling selective driver on the evolution of visual systems in Mysticeti, and the detection of schools of tiny prey at night would seem to be problematic without echolocation. The reliance of various mysticete species on RH1 might represent one solution for improved night vision given that rods are more useful than cones for contrast detection and hence picking out schools of prey from the background. Along these lines, the parallel pseudogenization of both cone opsins in Cetacea (Figure 1) could be the result of natural selection favoring an all-rod retina, in which case cone opsins were either selected against because of interference with RH1, or were rendered ‘jobless’ by the elimination of cones and released from selective constraints on color vision in this aquatic clade [22].

### Conclusions

The emergence of Cetacea represents a profound macroevolutionary transition that entails comprehensive remodeling at both the genetic and morphological levels [4]. Our results elucidate key events in the evolutionary history of cetacean opsins, including an initial blue shift of RH1 in stem Cetacea, parallel knockouts of *SWS1* in Odontoceti and Mysticeti, and five independent inactivations of *LWS* in deep-diving cetacean lineages. As correctly surmised by McFarland [20], some cetaceans are rod monochromats and have evolved eyes that are highly specialized for dim-light vision.

## Materials and Methods

### Taxon and Gene Sampling

Previously published *RH1*, *SWS1*, and *LWS* sequences for Cetacea were combined with new sequences that were generated through PCR and dideoxy sequencing. We targeted complete coding regions of all three opsin genes for representatives of Hippopotamidae and all extant cetacean families (Text S2). *RH1*, *LWS*, and *SWS1* sequences for additional cetartiodactyl families [Bovidae, Cervidae, Suidae, Camelidae] were assembled from Ensembl, Pre-Ensembl, and NCBI based on availability with minor augmentation by new sequences (Table S1, Text S2).

### PCR and Sequencing

Aligned sequences for *Bos taurus*, *Sus scrofa*, *Tursiops truncatus*, *Vicugna pacos*, and available GenBank sequences (Table S1) were used to design PCR primers for *SWS1*, *RH1*, and *LWS*. *SWS1* (exons 1–4; partial exon 5; introns 1–4) was amplified in five overlapping segments. PCR primers for *RH1* (exons 1–5) and *LWS* (exons 1–6) were positioned in the flanking intronic regions of each exon (see Text S2 for additional details on PCR reactions). Accession numbers for new cetartiodactyl sequences are KC676796–KC677023 (Table S1). Primer sequences are provided in Table S2.

### Alignments and Phylogenetic Analyses

Sequences were aligned manually using Se-Al [95]. The virtual mRNA alignment lengths were 1014 base pairs (bp) for *SWS1*, 1092 bp for *LWS*, and 1044 bp for *RH1*. The complete alignment for *SWS1*, including exons and introns, was 4163 bp. All alignments for phylogenetic and PAML analyses, along with alignments for non-overlapping PCR amplicons (exons plus partial introns for LWS and RH1), are provided in Text S3 in nexus format. Phylogenetic analyses were performed with RAxML 7.2.7 [96] and the GTR + Γ model of sequence evolution. Additional details are provided in Text S2.

### Inactivating Mutations

Opsin alignments were manually inspected for putative inactivating mutations, including substitutions that result in stop codons, changes at intron splice donor/acceptor sites, and frameshift indels. We also examined *SWS1* sequences for a missense mutation at Schiff's counterion site (E113G; bovine RH1 numbering) that disrupts opsin-chromophore binding [23].

### Ancestral Sequence Reconstructions and Character State Mapping

Ancestral DNA sequences for *SWS1*, *LWS*, and *RH1* were reconstructed with the Baseml program implemented in PAML 4.4b [45]. We used the REV model and a composite species tree based on McGowen et al. [42] for cetaceans and Gatesy [97] for all other cetartiodactyls. Frameshift mutations and other indels were optimized with Fitch parsimony, as implemented in Mesquite [98].

### *λ*_max_ Estimation

Spectral tuning in RH1 is influenced by at least 13 amino acid sites [28], although replacements at only three of these sites (83, 292, 299) fully explain the absorbance difference between cow RH1 (*Bos taurus*, λ_max_ = 500 nm) and bottlenose dolphin RH1 (*Tursiops truncatus*, λ_max_ = 488 nm) [99]. These replacements are D83N, A292S, and A299S. Different combinations of ancestral and derived amino acids at these three sites have been tested in mutagenic studies of *Bos* RH1 to explain the various λ_max_ values that occur in other cetaceans [21], [25]. For LWS, Yokoyama [36] suggested a “five-sites” rule whereby λ_max_ values between 510 and 560 in vertebrates can be fully explained by amino acid changes S180A, H197Y, Y277F, T285A, A308S and their interactions. Here, we follow Fasick et al. [21] and Bischoff et al. [25] and provide λ_max_ estimates for newly determined *RH1* and *LWS* sequences based on directly determined λ_max_ values from expressed RH1 and LWS pigments that possess identical amino acids at the same key sites for each of these opsins. It will be important in future studies to perform direct measurements of λ_max_ on reconstructions of ancestral RH1 sequences. Even without these experiments, empirical measurements on a diverse array of opsins from cetacean species and *Bos taurus* (wild type and mutagenesis variants) provide a strong foundation for inferring λ_max_ values in ancestral cetacean sequences [21], [25].

### dN/dS Analyses

The Codeml program in PAML 4.4b [45] was used to estimate the ratio (ω) of the non-synonymous substitution rate [dN] to the synonymous substitution rate (dS) at individual sites (*RH1*, *LWS*) and on branches (*SWS1*, *LWS*). We also performed branch-site analyses [45]–[47], [100] on *RH1* and *LWS* sequences. In both cases, branches with predicted changes in λ_max_ (Figure 1) of the relevant opsin were assigned to the foreground, and all other branches were assigned to the background. We used a composite species tree for all cetartiodactyl taxa as detailed above. Statistical tests of neutrality [complete absence of functional constraints] for branches and sets of branches were executed as in Meredith et al. [16]. See Text S2 for details.

## Supporting Information

Figure S1 Maximum likelihood phylogram based on *SWS1* exons and introns.
(PDF)

Figure S2 Maximum likelihood phylogram based on *SWS1* exons.
(PDF)

Figure S3 Maximum likelihood phylogram based on *RH1* exons.
(PDF)

Figure S4 Maximum likelihood phylogram based on *LWS* exons.
(PDF)

Figure S5 Parsimony reconstruction of the 4-bp frameshift deletion in *SWS1*. Branch colors are as follows: gray, odontocetes; black, mysticetes, blue, stem Cetacea; green, non-cetacean. Plio = Pliocene; P = Pleistocene. Paintings are by Carl Buell. Also see Text S1.
(PDF)

Figure S6 Chromatograms that illustrate inactivating mutations found in cetacean *LWS* sequences. Taxa exhibiting the deleterious mutations for the indicated exon are in red font. Deletions are highlighted in red and insertions are highlighted in green.
(PDF)

Table S1 Taxa and gene segments used in this study. * = new sequence; ^+^ = only sequenced exons 1–2, intron 1 and partial intron 2 to further delineate the distribution of inactivating mutations in *SWS1*. Gene and exon identities of new sequences are as follows: *LWS* Exon 1: KC676796–KC676816; *LWS* Exon 2: KC676817–KC676838; *LWS* Exon 3: KC676839–KC676859; *LWS* Exon 4: KC676860–KC676879; *LWS* Exon 5: KC676880–KC676899; *LWS* Exon 6: KC676900–KC676920; *RH1* Exon 1: KC676921–KC676938; *RH1* Exon 2: KC676939–KC676957; *RH1* Exons 3-4: KC676958–KC676977; *RH1* Exon 5: KC676978–KC676995; *SWS1* Exons 1-5: KC676996–KC677023. The amplicon containing *LWS* Exon 1 of *Eschrichtius* is shorter than 200 bp and could not be deposited in GenBank. The complete sequence can be found in Text S3.
(PDF)

Table S2 Primers used in this study (all 5′ to 3′). *SWS1* primer pairs were designed to amplify four complete exons (1–4), four complete introns (1–4), and part of exon 5 in five overlapping segments. *RHI* and *LWS* primers were designed to amplify each exon and part of the flanking 5′ and 3′ introns. Ex = exon.
(PDF)

Text S1 Additional details on the 4-bp frameshift deletion in *SWS1*.
(PDF)

Text S2 Additional Materials and Methods.
(PDF)

Text S3 Fourteen alignments in nexus format. Alignment 1 = *SWS1* Introns + Exons (RAxML); Alignment 2 = *SWS1* Exons (PAML, RAxML); Alignment 3 = *LWS* Exons (PAML, RAxML); Alignment 4 = *LWS* Exon 1 amplicon; Alignment 5 = *LWS* Exon 2 amplicon; Alignment 6 = *LWS* Exon 3 amplicon; Alignment 7 = *LWS* Exon 4 amplicon; Alignment 8 = *LWS* Exon 5 amplicon; Alignment 9 = *LWS* Exon 6 amplicon; Alignment 10 = *RH1* Exons (PAML/RAxML); Alignment 11 = *RH1* Exon 1 amplicon; Alignment 12 = *RH1* Exon 2 amplicon; Alignment 13 = *RH1* Exons 3+4 amplicon; Alignment 14 = *RH1* Exon 5 amplicon.
(TXT)
