# Supplementary figures and images for: Rod Monochromacy and the Coevolution of Cetacean Retinal Opsins
Source: PLoS Genet. 2013 Apr 18;9(4):e1003432. doi: 10.1371/journal.pgen.1003432 (PMC3630094; doi:10.1371/journal.pgen.1003432)

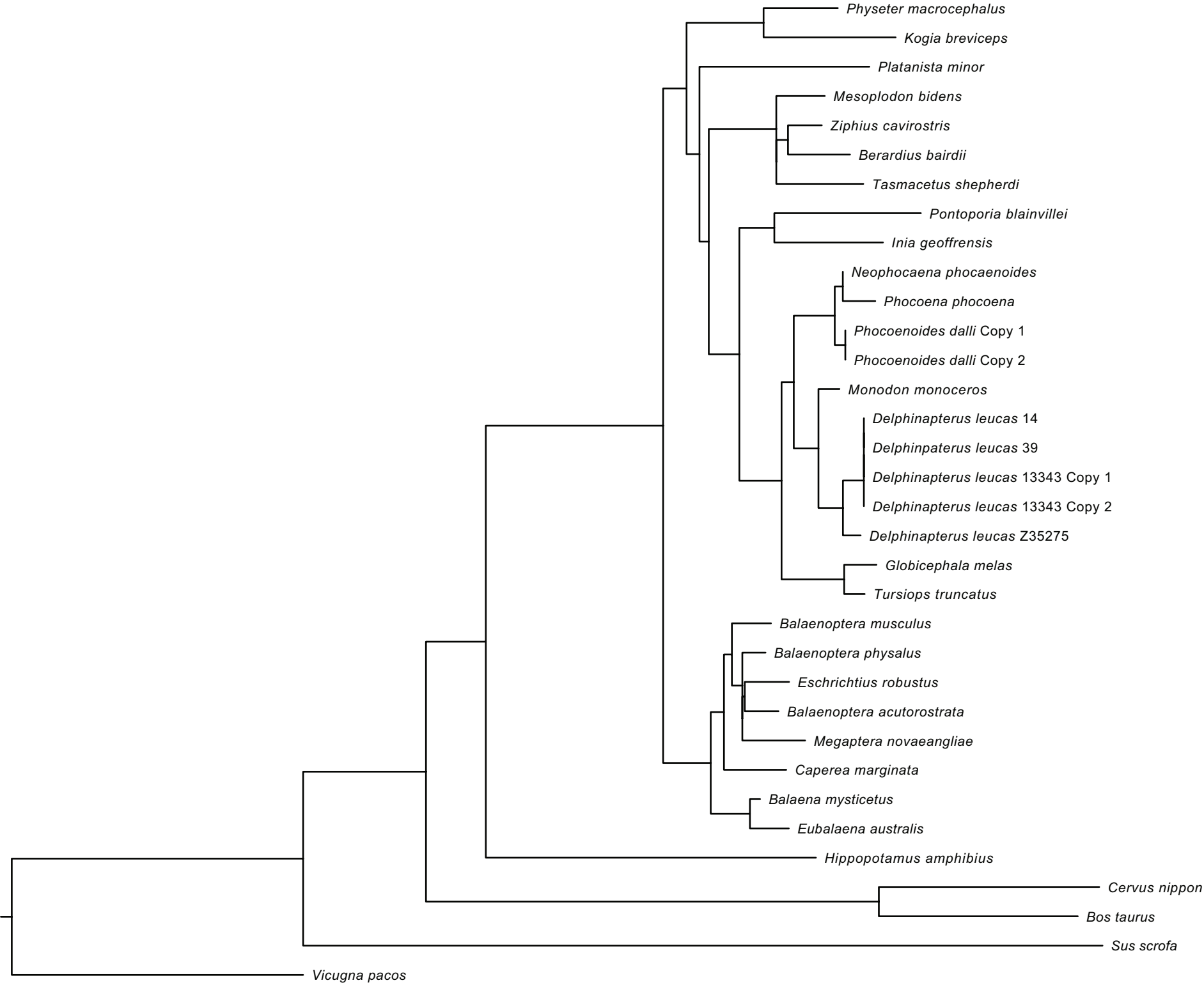

Supplement: Figure S1 — Maximum likelihood phylogram based on SWS1 exons and introns. (PDF) [file pgen.1003432.s001.pdf]

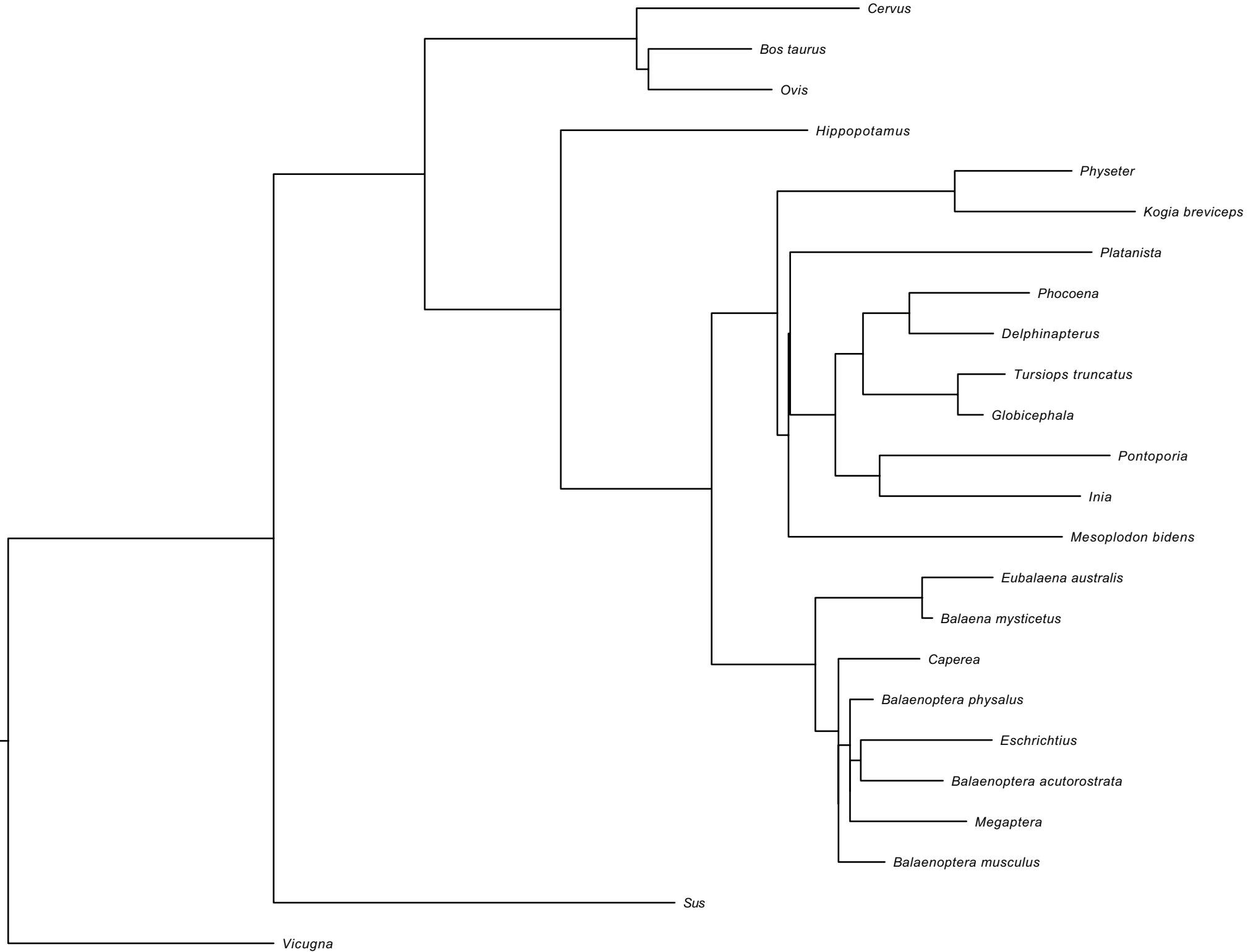

Supplement: Figure S2 — Maximum likelihood phylogram based on SWS1 exons. (PDF) [file pgen.1003432.s002.pdf]

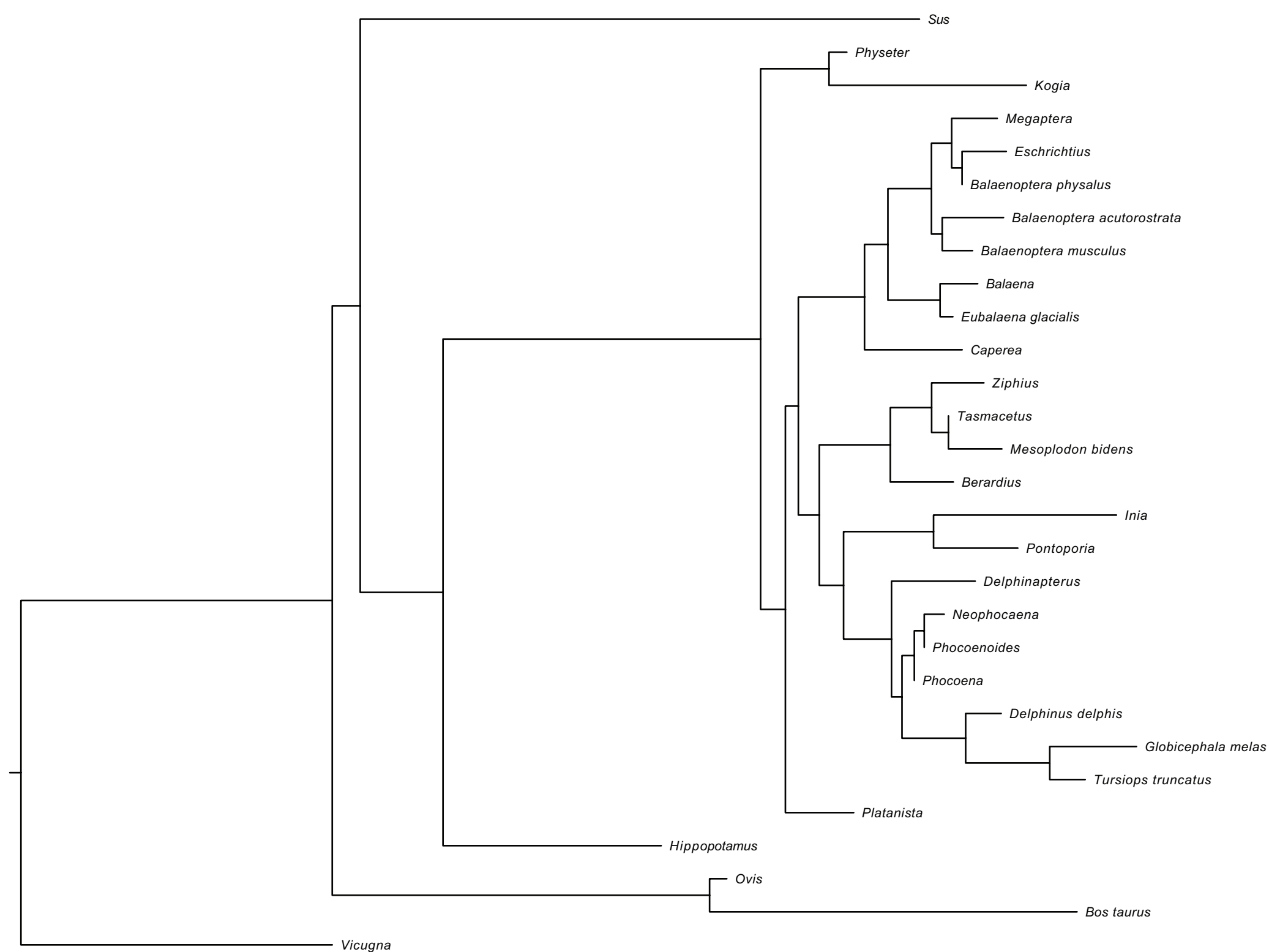

Supplement: Figure S3 — Maximum likelihood phylogram based on RH1 exons. (PDF) [file pgen.1003432.s003.pdf]

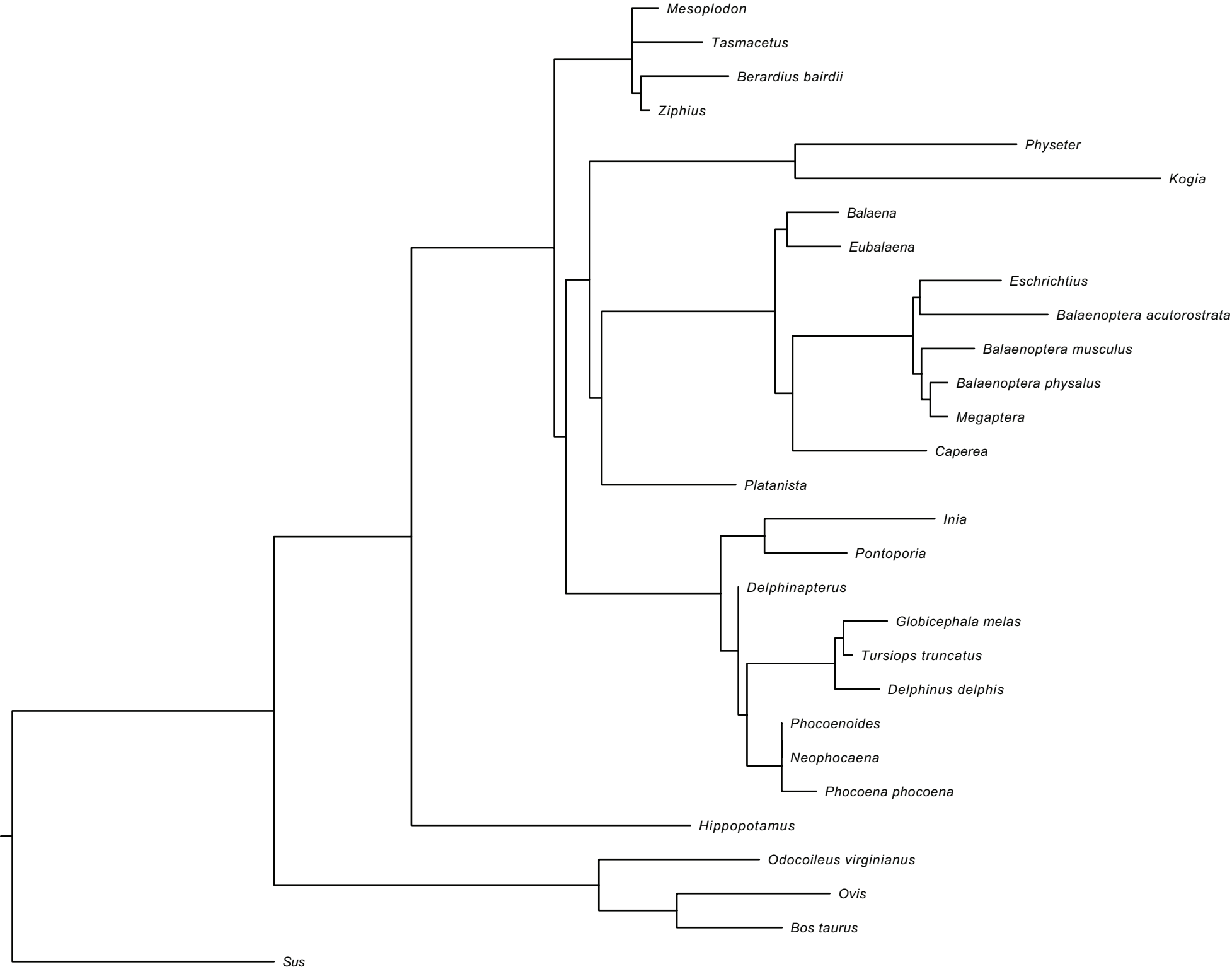

0.02

Supplement: Figure S4 — Maximum likelihood phylogram based on LWS exons. (PDF) [file pgen.1003432.s004.pdf]

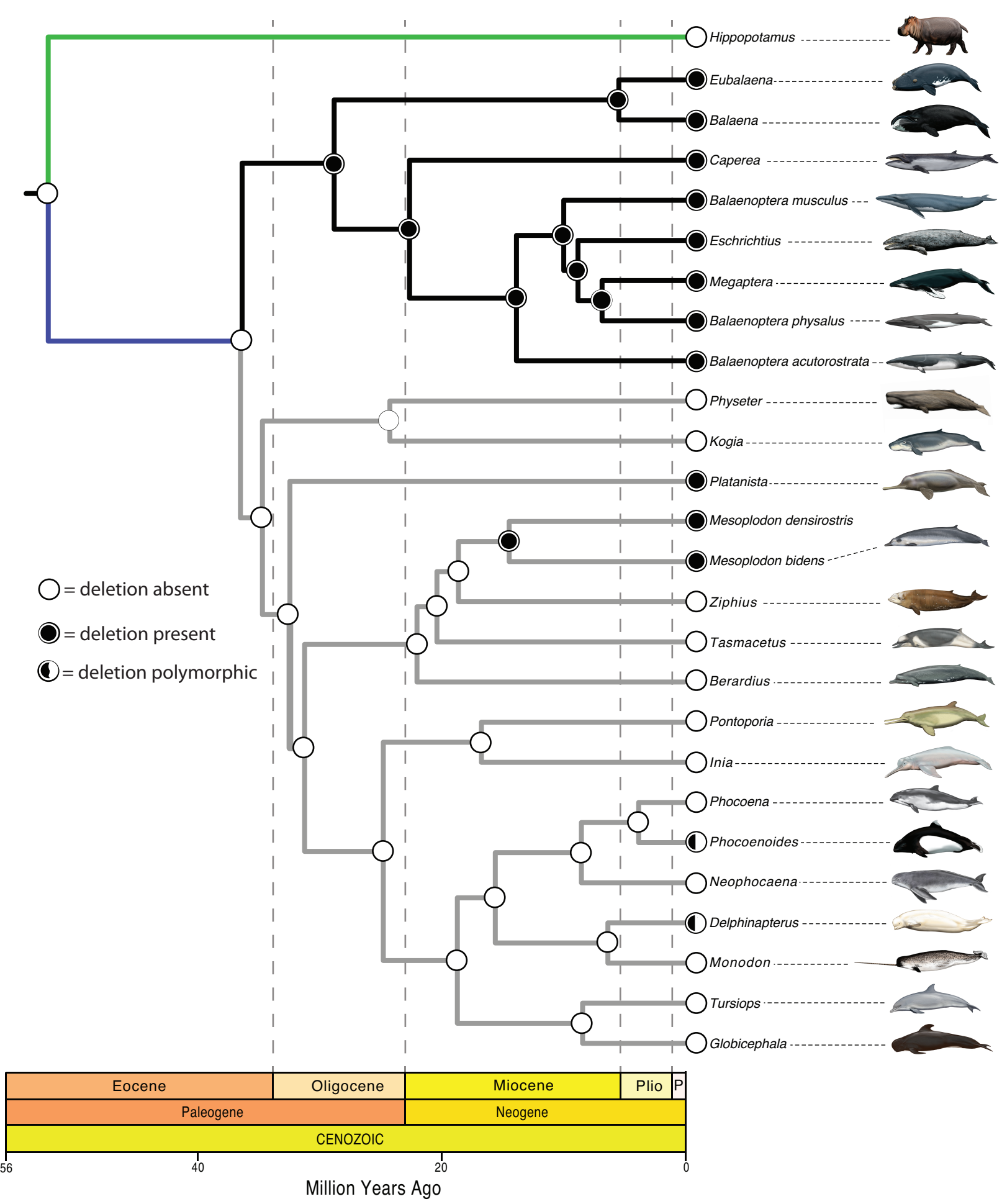

Supplement: Figure S5 — Parsimony reconstruction of the 4-bp frameshift deletion in SWS1. Branch colors are as follows: gray, odontocetes; black, mysticetes, blue, stem Cetacea; green, non-cetacean. Plio = Pliocene; P = Pleistocene. Paintings are by Carl Buell. Also see Text S1. (PDF) [file pgen.1003432.s005.pdf]

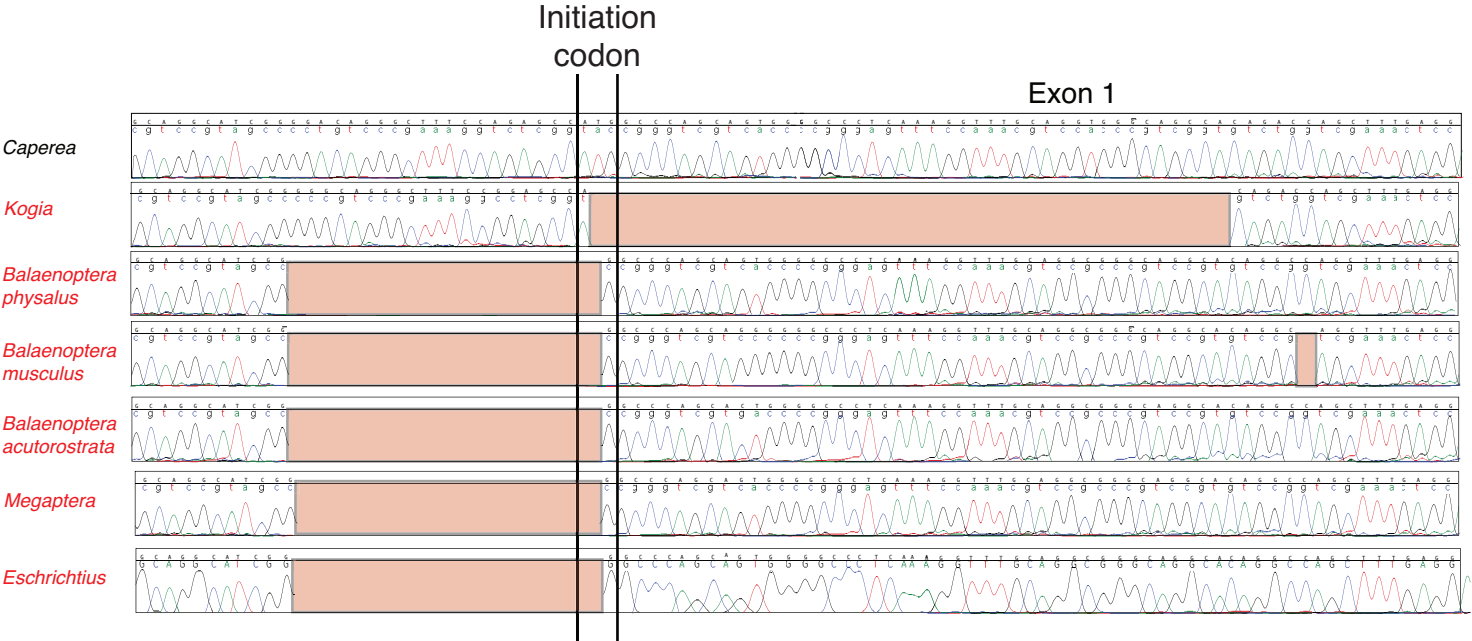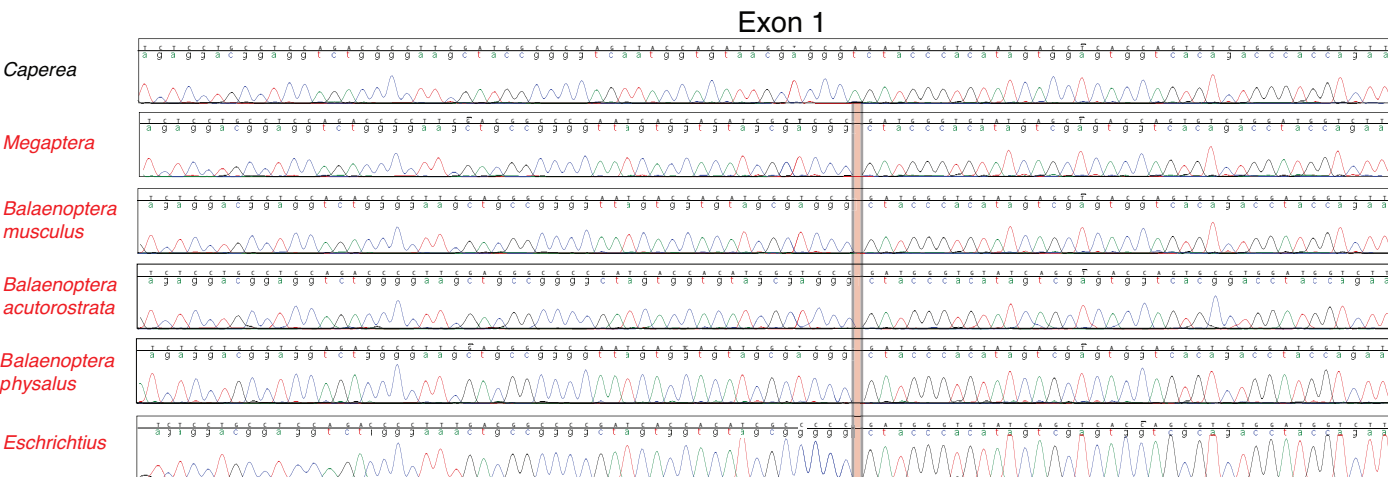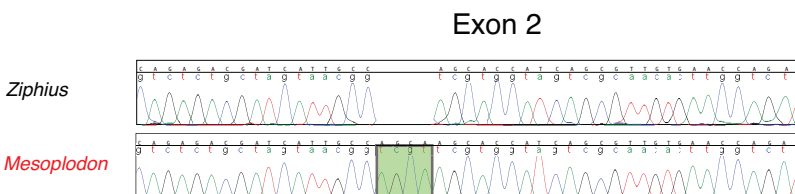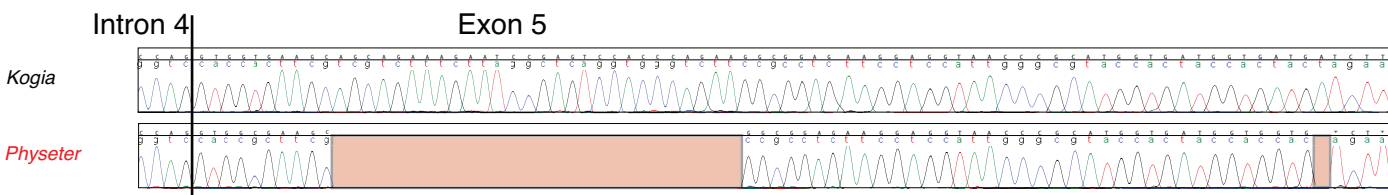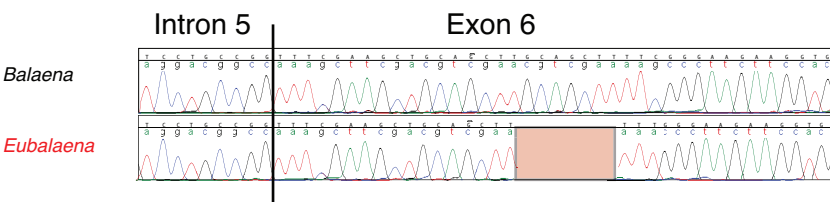

Supplement: Figure S6 — Chromatograms that illustrate inactivating mutations found in cetacean LWS sequences. Taxa exhibiting the deleterious mutations for the indicated exon are in red font. Deletions are highlighted in red and insertions are highlighted in green. (PDF) [file pgen.1003432.s006.pdf]
