# Supplementary material for: Rod Monochromacy and the Coevolution of Cetacean Retinal Opsins
Source: PLoS Genet. 2013 Apr 18;9(4):e1003432. doi: 10.1371/journal.pgen.1003432 (PMC3630094; doi:10.1371/journal.pgen.1003432)
Supplement: Table S1 — Taxa and gene segments used in this study. * = new sequence; + = only sequenced exons 1–2, intron 1 and partial intron 2 to further delineate the distribution of inactivating mutations in SWS1. Gene and exon identities of new sequences are as follows: LWS Exon 1: KC676796–KC676816; LWS Exon 2: KC676817–KC676838; LWS Exon 3: KC676839–KC676859; LWS Exon 4: KC676860–KC676879; LWS Exon 5: KC676880–KC676899; LWS Exon 6: KC676900–KC676920; RH1 Exon 1: KC676921–KC676938; RH1 Exon 2: KC676939–KC676957; RH1 Exons 3-4: KC676958–KC676977; RH1 Exon 5: KC676978–KC676995; SWS1 Exons 1-5: KC676996–KC677023. The amplicon containing LWS Exon 1 of Eschrichtius is shorter than 200 bp and could not be deposited in GenBank. The complete sequence can be found in Text S3. (PDF) [file pgen.1003432.s007.pdf]

Table S1

Cetartiodactyla Taxa	Retinal Opsin Gene		
	SWS1	*LWS*	*RH1*
Balaenidae			
Eubalaena australis (southern right whale)	*	*	
Eubalaena glacialis (North Atlantic right whale)			JQ730751
Balaena mysticetus (bowhead whale)	*	*	*
Neobalaenidae			
Caperea marginata (pygmy right whale)	*	*	*
Eschrichtiidae			
Eschrichtius robustus (grey whale)	*	*	*
Balaenopteridae			
Balaenoptera acutorostrata (common minke whale)	*	*	*
Balaenoptera physalus (fin whale)	*	*	*
Balaenoptera musculus (blue whale)	*	*	*
Megaptera novaeangliae (humpback whale)	AY228440	*	*
Delphinidae			
Globicephala melas (long-finned pilot whale)	AY228442	AY228446	AF055315
Tursiops truncatus (common bottlenose dolphin)	Ensembl 58	AF055457	AF055456
Delphinus delphis (short-beaked common dolphin)		AY228451	AF055314
Monodontidae			
Delphinapterus leucas Z35275 (beluga)	*	*	*
Delphinapterus leucas SWFSC Z13343 (beluga)	*		
Delphinapterus leucas NYSZ 14 (beluga)	*		
Delphinapterus leucas NYSZ 39 (beluga)	*		
Monodon monoceros (narwhal)	*		
Phocoenidae			
Phocoena phocoena (harbor porpoise)	*	AY228450	*
Neophocaena phocaenoides (finless porpoise)	*	*	*
Phocoenoides dalli (Dall's porpoise)	*	*	*
Iniidae			
Inia geoffrensis (Amazon River dolphin)	*	*	*
Pontoporiidae			
Pontoporia blainvillei (La Plata dolphin)	*	*	*
Platanistidae			
Platanista minor (Indus River dolphin)	*	*	*
Physeteridae			
Physeter macrocephalus (giant sperm whale)	*	*	*
Kogiidae			
Kogia breviceps (pygmy sperm whale)	*	*	*
Ziphiidae			
Mesoplodon bidens (Sowerby's beaked whale)	*	*	AF055316
Mesoplodon densirostris (Blainville's beaked whale)	AY228441		
Berardius bairdii (Baird's beaked whale)	*	*	*
Tasmacetus shepherdi (Shepherd's beaked whale)	*	*	*
Ziphius cavirostris (Cuvier's beaked whale)	*	*	*
Hippopotamidae			
Hippopotamus amphibius (hippopotamus)	*	*	*
Cervidae			
Cervus nippon (sika deer)	*		
Odocoileus virginianus (white-tailed deer)		AF132041	

Bovidae			
Bos taurus (cow)	Ensembl 58	NM_174566	NM_001014890
Ovis aries (sheep)		PreEnsembl	PreEnsembl
Suidae			
Sus scrofa (domestic pig)	Ensembl 58	NM_001011506	NM_214221
Camelidae			
Vicugna pacos (alpaca)	Ensembl 58		Ensembl 66
