# Supplementary material for: Rod Monochromacy and the Coevolution of Cetacean Retinal Opsins
Source: PLoS Genet. 2013 Apr 18;9(4):e1003432. doi: 10.1371/journal.pgen.1003432 (PMC3630094; doi:10.1371/journal.pgen.1003432)
Supplement: Table S2 — Primers used in this study (all 5′ to 3′). SWS1 primer pairs were designed to amplify four complete exons (1–4), four complete introns (1–4), and part of exon 5 in five overlapping segments. RHI and LWS primers were designed to amplify each exon and part of the flanking 5′ and 3′ introns. Ex = exon. (PDF) [file pgen.1003432.s008.pdf]

Table S2

LWS-Ex1Cet-IF3	GGTTTGGGAGCTTTTAAGGTGARGAG
LWS-Ex1Cet-IF4	GGCTGGTATAAAGCRCCGTGACC
LWS-Ex1Cet-IR1	CCATTCATGTTATCATAAAGGCTAMG
LWS-Ex1Cet-IR2	CGATATTACGGTGTCCTTTTAYATTCAG
LWS-Ex1Cet-IR3	CAGCCTTWGCACCTAYTCGCTCAC
LWS-Ex1Cet-IR4	GCCAGCRTGACCCAGCCTTWGCAC
LWS-Ex1Cet-IR5	GGAAGCCCATGTGGTTTGAAARTG
LWS-Ex1Cet-IR6	CTCAAAGGGTCTGGGAAGCCCATG
LWS-Ex1CetF1	GGARGTCTAAGTCCCAGGCCCAATTAAGAG
LWS-Ex1CetF2	GGCCCAATTAAGAGATCAGATGGYGAARGG
LWS-Ex1CetR1	CCTRACTCRTCTTTGCRTCTCCCAAAGCC
LWS-Ex1CetR2	GGTGAGTGMCTTCGAGAGCCTAAGG
LWS-Ex2Cet-IF1	GACCAGTCGCAAAGGTGAGTGMGGC
LWS-Ex2Cet-IF2	GGCTGAGGGGAGATTCTGATGGGGYG
LWS-Ex2Cet-IF3	GGGCGGCACAGATGTAGCTGTSC
LWS-Ex2Cet-IF4	CNCTSTGTGTCTCCTGCCTCCAG
LWS-Ex2Cet-IR3	GTGCCCAGGCCCCGGCTGGCTTAC
LWS-Ex2CetF1	GCYTTGTTAAGTTCAAGGATGCCCRTGCAGG
LWS-Ex2CetF2	GCAGGYCGTGGCCTCGRGYTTGAGAGAC
LWS-Ex2CetR1	CAGCCTSCCGCRCGTAAAGGARACMCTCATG
LWS-Ex2CetR2	GGCAAAATCMGCAGAKGTAGCTSTGGG
LWS-Ex3Cet-IR1	GCTGCYCCTGTTTGGGAGAAACAGAC
LWS-Ex3Cet-IR2	CCCTCAGCAGGTGCAAGAAAGAGGC
LWS-Ex3Cet-IR3	GCCTTCCTGGCACCCTGGCATCCTTAC
LWS-Ex3Cet-IR4	GYTCTAAACKAATCGCCTTCCTGGCACCC
LWS-Ex3Cet-IR5	GGGAGAAACAGACATTTGCAGAGGCTTGG
LWS-Ex3Cet-IR6	GCTGYTCCTGTTTGGGAGAAACAGAC
LWS-Ex3CetF1	CAGGKGTTAGAGAAAGAAACTGACCAAGAG
LWS-Ex3CetF2	GTTGMCTCAAAGCCCTCCTMTGTCTGC
LWS-Ex3CetR1	GGTGAYGGATGCACAACAYTGTGAATG
LWS-Ex3CetR2	CAACAYTGTGAATGYACCAAACCTC
LWS-Ex4Cet-IF1	GGTTAATTCATGTAAYGTGMATTTC
LWS-Ex4Cet-IF2	GCCCAACAACCTTACCCAAGTCMGTGG
LWS-Ex4Cet-IF3	CCTTACCCAAGTCMGTGGTGTGCTGG
LWS-Ex4Cet-IR1	GCCTAGACCTCCCYGGCGTTCAYG
LWS-Ex4Cet-IR2	GGTCCTYTGTGCCAGCCTAGACCTCC
LWS-Ex4CetF1	GGTGAYGGATGCACAACAYTGTGAATG
LWS-Ex4CetF2	CAACAYTGTGAATGYACCAAACCTC
LWS-Ex4CetR1	CACAGAGGACCGCMCAGTCYGCTCTCC

LWS-Ex4CetR2	CAGTCYGCTCTCCWGCCTCMAAGCCAG
LWS-Ex5Cet-IF1	GGCCCATCTGCTTCTCCCCRCTACCCTCC
LWS-Ex5Cet-IF2	CCCCRCTACCCTCCCTGACTAGCTAC
LWS-Ex5Cet-IF3	GAATCCCCTGGGGTACGGAGCCCTGAGG
LWS-Ex5Cet-IF4	GGCSACTCTCTGTCYCCCCCAG
LWS-Ex5Cet-IR1	GCTCTGTCAACTGCGTGACTTA
LWS-Ex5Cet-IR2	CCATCTGCAGTTTGCTCTGTCAACTG
LWS-Ex5CetF1	CTTTCCTCTACRAAGCRGCTTGTGCCCAC
LWS-Ex5CetF2	GCTTGTGCCCACAATCGGMTCAGC
LWS-Ex5CetR1	CAAACTRCAGATGGACCCYGGAAGAGC
LWS-Ex5CetR2	GACCCYGGAAGAGCCCAGTKATGGCTCCC
LWS-Ex6Cet-IF1	GCTCTGCTCARTRACACTTTCTGTCC
LWS-Ex6Cet-IF1	GCTCTGCTCARTRACACTTTCTGTCC
LWS-Ex6Cet-IR1	GTTACGTTKTCCAGTGGGAGACAGRCGAC
LWS-Ex6Cet-IR1	GTTACGTTKTCCAGTGGGAGACAGRCGAC
LWS-Ex6Cet-IR2	CTCGGTTGCTTTYGGCAAAGAGAAACTAC
LWS-Ex6Cet-IR3	GTGGCAGCCTCTATGACTCGGTTGCTTTYGGC
LWS-Ex6CetF1	CATTTAGCCTAGCAYGCTGGCCCTRCC
LWS-Ex6CetF2	CAYGCTGGCCCTRCCTGCTCTGC
LWS-Ex6CetR1	GAGWTGYTCATCTTTTMTAAGCCTCSTGTC
LWS-Ex6CetR2	GGAGCTAGCRAAGCCAGGCCCTTTAAGGC
RH1-Ex1Cet-IF1	CAGAGCCTGGAGYCATCCAGCCGGAGCCC
RH1-Ex1Cet-IF2	CCAGCCGGAGCCCTGYGTGGCTGAGCTC
RH1-Ex1Cet-IR1	CCYGTCACCCGCACCCCAGCTCATAC
RH1-Ex1Cet-IR2	CCTGGCTCCTGTAGCCCCYGTCACCCGCACC
RH1-Ex1F1	GTGRGGATTAATATGATTAATAAYGCC
RH1-Ex1F2	CCCAATCKCSCAGGTGCTGATTCAGCC
RH1-Ex1R1	CCMCCAYCCCCAAGGCHGRGGCTG
RH1-Ex1R2	CCCTCVACAAACGWCAGCCKAGC
RH1-Ex2Cet-IF1	GGTCCAGCCACGTGGCRTGTTGACTGAC
RH1-Ex2Cet-IF2	GGCRTGTTGACTGACAGCTGGCTRCCTTGC
RH1-Ex2Cet-IR1	GTCYCCTTGACATGGGGACCAGCCCCAYAGC
RH1-Ex2Cet-IR2	GGGGTGTCTTGGAGTCYCCTTGACATGG
RH1-Ex2F1	CYCAKGTTGTTACATKTCCCTGGARG
RH1-Ex2F2	CAGCCRTGACCCCGYGACMCGATTKCTGTG
RH1-Ex2R1	GCCAAGGTGAAWGGTTGGGGYGTCTTGG
RH1-Ex2R2	GTTGVGGATTAGGGGCACAGCCAAGGTG
RH1-Ex3-4Cet-IF1	GACTCTGKTCAGCTGGGCCTGTTCCCARG
RH1-Ex3-4Cet-IF2	CCTGTTCCCARGTCCCTCACAYGTGGGTCTCCC
RH1-Ex3-4Cet-IF3	CATMTTCTTCTGCTACGGGCAGCTGGTC
RH1-Ex3-4Cet-IF4	GGGCAGCTGGTCTTCACAGTCAAGGAG

RH1-Ex3-4Cet-IR1	CCATGCGAGTGACCTCCTTCTCGGCCTTC
RH1-Ex3-4Cet-IR2	CCATGATGAYGACCATGCGAGTGACCTCC
RH1-Ex3-4Cet-IR3	GGACCCTCCCGCCYCACCAGCAGGCAC
RH1-Ex3-4Cet-IR4	GTGGCCTTGGGGGACCYGGACCCTCCCGCC
RH1-Ex3-4F1	CAGKGAMACAACACTGTTTSCAC
RH1-Ex3-4F2	GYGAGGCCCAGAGTGAGASCAGCC
RH1-Ex3-4R1	CCATTTTGCAGGTGGAASGACBGARG
RH1-Ex3-4R2	CCAGGARTCTGMATTTCTCCCAGATGAC
RH1-Ex5Cet-IF1	GGACCCTGACACTGACTCATGCC
RH1-Ex5Cet-IF2	CTGACTCATGCCCTTYCTTCCAG
RH1-Ex5Cet-IR1	CCTACAGTTGGCCACAGAGTC
RH1-Ex5Cet-IR2	GGGAGACTCCTACAGTTGGCC
RH1-Ex5F1	GCTGTGTGRCCTTGGGCAAGTMACTTCCC
RH1-Ex5F2	CAAGTMACTTCCCTTCTYTGGGCWTCAG
RH1-Ex5R1	CCTGTGSGAWCTGGTTCYGACAGG
RH1-Ex5R2	ACTCAGGGAWCCTGTGSGAWCTGG
SWS1-CetF4	GGCCTTCTTGGBCTTTGAGCGCTACATYATC
SWS1-CetF1	GGGAGGATRATCTATAAGAGGAATCAG
SWS1-CetF10	CWAGGAGGGTTCCTGTCKTVTAGRGC
SWS1-CetF2	CTATAAGAGGAATCAGAGRGGGTGGKGGACATCC
SWS1-CetF3	CAGGCTGGTCMCTGGCCTTCTTGGBCTTTG
SWS1-CetF5	CYGTGGGCACCAARTAYTACAGCGAG
SWS1-CetF6	CCAARTAYTACAGCGAGTAYTATACCTGG
SWS1-CetF7	GCTGCCCTKGCCATGTATATAGTCAACAACC
SWS1-CetF8	GTATATAGTCAACAACCRTAACCABGGG
SWS1-CetF9	AACTCAGAYAAATCWAGGAGGGTTCCT
SWS1-CetIF1	GRGGCCTTCCTGGGCHGTRCAGCAGGTA
SWS1-CetIF2	GGGAGACAAGKGCTAAAGTTTACYCTG
SWS1-CetIF3	GAGAGCTGSCTCCATCTYGCCTTCCTCAA
SWS1-CetIF4	GGACTCYACTCWAAGGSTTTCTAGKGG
SWS1-CetIF5	GRGTYAGCCAGAATGTGYAGGGATTA
SWS1-CetIR1	GRTDGGAACCAGGGRCCAGKCTGTTGG
SWS1-CetIR2	GTTCTAAGHGGAGAACACAAYC
SWS1-CetIR3	GGGACTAGGCCAGAAGSTGGGACAGMAGAGA
SWS1-CetIR4	GTAAGCMCAGGGATGAAGGYGTGGTC
SWS1-CetIR5	CTTCCTGABGTKATCTCCTCKGTAC
SWS1-CetR1	GYGTCTCCATCCCACCSTTCTTTGGCTG
SWS1-CetR10	CTACYGTCTCTTCTAGCCAAGTTGGCCCC
SWS1-CetR2	CCATCCCACCCTTCTTTGGCTGGAGCCG
SWS1-CetR3	CATCTGCTTCTCCTACTCTCAGCTGCTGGGG
SWS1-CetR4	CCTACTCTCAGCTGCTGGGGGCCYTCAGAGC

SWS1-CetR5	CTTGYATCTACAATCCSATCATCTACTGC
SWS1-CetR6	CCSATCATCTACTGCTTCATGAATAAGCAG
SWS1-CetR7	GMATTGGCAGTCCTTTCCCAGTTGGRAGC
SWS1-CetR8	CAGTCCTTTCCTAGTTGGRAGCACYTTC
SWS1-CetR9	GAAGTKTCTACYGTCTCTTCTAGCC
