# Supplementary material for: Rod Monochromacy and the Coevolution of Cetacean Retinal Opsins
Source: PLoS Genet. 2013 Apr 18;9(4):e1003432. doi: 10.1371/journal.pgen.1003432 (PMC3630094; doi:10.1371/journal.pgen.1003432)
Supplement: Text S1 — Additional details on the 4-bp frameshift deletion in SWS1. (PDF) [file pgen.1003432.s009.pdf]

Text S1. Additional details on the 4-bp frameshift deletion in *SWS1*.

Our new cetacean *SWS1* sequences were virtually identical to all previously reported Genbank sequences for overlapping regions/taxa. However, our *Delphinapterus* sequence has a 4-bp frameshift deletion that is located in the same alignment position as the 4-bp deletion previously reported in all baleen whales, *Mesoplodon*, and *Platanista* [1]. Levenson and Dizon [1] sequenced three *Delphinapterus* individuals but none of them had the 4 bp deletion. To understand the phylogenetic distribution of the 4 bp deletion within odontocetes, we sequenced additional species/individuals for the *SWS1* region containing the 4 bp deletion (~800 bp: exon 1, intron 1, partial exon 2). The additional sequences included three *Delphinapterus* individuals (SWFSC Z13343, NYZS 14, NYZS 39), *Monodon monoceros* (Monodontidae; narwhale), *Neophocaena phocaenoides* (Phocoenidae; finless porpoise), *Phocoenoides dalli* (Phocoenidae; Dall's porpoise), and the additional beaked whales (Ziphiidae) *Berardius bairdii* (Baird's beaked whale), *Tasmacetus shepherdi* (Shepherd's beaked whale), and *Ziphius cavirostris* (Cuvier's beaked whale).

The 4-bp deletion was absent in *Monodon*, *Tasmacetus*, *Berardius*, *Ziphius, Neophocaena,* and one of the three *Delphinapterus* individuals (NYZS 14). In *Delphinapterus* (SWFSC Z13343) and in *Phocoenoides* there are two *SWS1* copies (presumably alleles), one with the 4-bp deletion and the other without this frameshift (Figure S5). A ML phylogenetic analysis that included the additional partial *SWS1* sequences (GTR + Γ model of sequence evolution, 500 bootstrap replicates, randomized MP starting trees, the fast hill-climbing algorithm, with all other free parameters estimated) recovered all of the *Delphinapterus* specimens as monophyletic, and the two alleles from *Phocoenoides* also clustered together in the tree. A parsimony reconstruction of the 4-bp frameshift deletion in *SWS1* is shown in Figure S5. Phylogenetic relationships and divergence times in Figure S5 follow McGowen et al. [2].

References

1. Levenson DH, Dizon A (2003) Genetic evidence for the ancestral loss of SWS cone pigments in mysticete and odontocete cetaceans. Proc Roy Soc London B 270: 673-679.

2. McGowen MR, Spaulding M, Gatesy J (2009) Divergence date estimation and a comprehensive molecular tree of extant cetaceans. Mol Phylogenet Evol 53: 891-906.
