# Supplementary material for: Rod Monochromacy and the Coevolution of Cetacean Retinal Opsins
Source: PLoS Genet. 2013 Apr 18;9(4):e1003432. doi: 10.1371/journal.pgen.1003432 (PMC3630094; doi:10.1371/journal.pgen.1003432)
Supplement: Text S2 — Additional Materials and Methods. (PDF) [file pgen.1003432.s010.pdf]

Text S2. Additional Materials and Methods.

Taxon and Gene Sampling. Complete coding regions of *RH1* and *LWS* were targeted for representatives of outgroups and extant cetacean families as follows (DNA sources/specimen numbers in brackets): Camelidae (*Vicugna pacos* [Ensembl]), Suidae (*Sus scrofa* [Ensembl, GenBank]), Bovidae (*Bos taurus* [Ensembl, GenBank], *Ovis aries* [PreEnsembl]), Cervidae (*Cervus nippon* [M. Cronin], *Odocoileus virginianus* [GenBank]), Hippopotamidae (*Hippopotamus amphibious* [W. Murphy HAM0003-DN-01]), Balaenidae (*Balaena mysticetus* [SWFSC Z6985 from NSB], *Eubalaena australis* [SWFSC Z18928 from SAM]), Neobalaenidae (*Caperea marginata* [Ú. Árnason]), Eschrichtiidae (*Eschrichtius robustus* [SWFSC Z5750]), Balaenopteridae (*Balaenoptera acutorostrata* [SWFSC Z13091 from TMMC], *B. musculus* [SWFSC Z4502], *B. physalus* [H. Rosenbaum], *Megaptera novaeangliae* [H. Rosenbaum 6AO2-602]), Physeteridae (*Physeter macrocephalus* [M. Milinkovitch]), Kogiidae (*Kogia breviceps* [SWFSC Z10119]), Platanistidae (*Platanista minor* [SWFSC Z15224 from G. Braulik, WWF]), Ziphiidae (*Berardius bairdii* [SWFSC Z4963 from LACM], *Mesoplodon bidens* [SWFSC Z3859], *Tasmacetus shepherdi* [SWFSC Z4971 from SIDOM], *Ziphius cavirostris* [SWFSC Z2157]), Pontoporiidae (*Pontoporia blainvillei* [SWFSC Z9834]), Iniidae (*Inia geoffrensis* [SWFSC Z505 from SIDOM]), Monodontidae (*Delphinapterus leucas* [SWFSC Z35275 from AKDFG]), Phocoenidae (*Neophocaena phocaenoides* [SWFSC 9559], *Phocoena phocoena* [SWFSC Z28452 from TMMC], *Phocoenoides dalli* [SWFSC 38979]), Delphinidae (*Delphinus delphis* [GenBank], *Globicephala melas* [GenBank], *Tursiops truncatus* [Ensembl, GenBank]). Complete protein coding and intronic regions of *SWS1* were targeted for all of the above taxa, except for one delphinid (*Delphinus delphis*), three ziphiids (*B. bairdii*, *T. shepherdi*, *Z. cavirostris*) and two phocoenids (*N. phocaenoides, P. dalli*). For the three ziphiids and two phocoenids listed above, PCR was limited to the region that contains a 4-bp deletion (~800 bp: exon 1, intron 1, partial exon 2) that was previously identified by Levenson and Dizon [1]. *Monodon monoceros* [SWFSC Z8260 from M. P. Heide-Jørgensen, GINR] and three additional *Delphinapterus* individuals (SWFSC Z13343 from NMFS NMML, NYZS 14, NYZS 39) were also targeted for this ~800 bp region of *SWS1*. Abbreviations: SWFSC =

Southwest Fisheries Science Center; SAM = South Australian Museum; NSB = North Slope Borough, Barrow, Alaska, USA; TMMC = The Marine Mammal Center – Sausalito; GINR = Greenland Institute of Natural Resources; AKDFG = Alaska Department of Fish and Game; SIDOM = Smithsonian Institution Division of Mammals; NYZS = New York Zoological Society; WWF = World Wildlife Fund; LACM = Los Angeles County Museum; NMFS NMML = National Marine Fisheries Service, National Marine Mammal Laboratory.

PCR Reactions. PCR reactions were carried out using Denville Scientific Inc. Ramp-Taq DNA polymerase in 50 μl reactions with the following thermal cycling parameters: initial denaturation at 95°C for 7 minutes; 35 cycles of 1 minute at 95°C (denaturation), 1 minute at 50°C (annealing), and 1 minute at 72°C (extension); and a final extension at 72°C for 10 minutes. Nested PCR reactions were performed using the aforementioned PCR regime if the primary PCR reaction failed to yield a usable product. One μl of the original PCR product was used as the template DNA in the nested PCR reactions. PCR products were run out on 1% agarose gels, excised, and cleaned with Bioneer AccuPrep Gel Purification Kits. Cleaned PCR products were sequenced in both directions using an automated DNA sequencer (ABI 3730xl) at the UCR Core Instrumentation Facility. Contigs were assembled using Sequencher 4.8.

Phylogenetic Analyses. ML analyses with RAxML 7.2.7 [2] searched for the best-scoring ML tree in a single run, employed 500 bootstrap replicates, randomized MP starting trees, the fast hill-climbing algorithm, with all other free parameters estimated. For the combined *SWS1* data set, unique model parameters (GTR + Γ) were permitted for exons and for introns.

dN/dS Analyses. Site, branch, and branch-site analyses were performed with two different codon frequency models (CodonFreq = 2 and 3) in PAML [3]. Site analyses were performed to test whether a subset of sites have evolved under positive selection in conjunction with the deployment of cetaceans into different aquatic habitats. Site analyses were performed with model 1 (null) versus model 2 and model 8a (null)

versus model 8. Site and branch-site analyses with *LWS* were executed after pruning taxa with frameshift mutations in this gene. Site and branch-site analyses with *RH1* were executed after excluding four incomplete sequences (*Tasmacetus*, *Eschrichtius*, *Ovis*, *Vicugna*) that resulted in computational errors with codeml. Sites analyses were not performed on *SWS1* because *SWS1* is inactivated in all crown cetaceans. Branch analyses were performed on *SWS1* and *LWS* to elucidate the history of purifying selection on branches that pre-date the occurrence of inactivating mutations. Branches were binned into functional, pre-mutation, transitional, and pseudogenic categories following Meredith et al. [4]. Branch-site analyses on *RH1* and *LWS* were performed with a modified version of model A and the corresponding null model [3, 5, 6]. The null distribution is a 50:50 mixture of point mass 0 and X^2 with one degree of freedom, which yields critical values 2.71 at 5% and 5.41 at 1%. However, we followed Yang [3] and calculated P values with X^2 and one degree of freedom (i.e., no 50:50 mixture) to guard against violations of model assumptions. Two *RH1* sequences (*Tursiops truncatus* [AF055456], *Globicephala melas* [AF055315]) have an eight-bp region, which is embedded in the codons that encode amino acids 194-196, that is out of alignment with other cetaceans owing to an apparent one-bp deletion that is compensated by a one-bp insertion. However, the corresponding Ensembl sequence for *T. truncatus* lacks this anomalous feature. Given the possibility of sequencing error in the GenBank sequences, we coded this three-codon region as missing in dN/dS analyses with *RH1*. The amino acid replacements at sites 194 and 195 in *T. truncatus* and *G. melas*, whether real or spurious, also account for half of the replacements within Cetacea at these sites.

References

1. Levenson DH, Dizon A (2003) Genetic evidence for the ancestral loss of SWS cone pigments in mysticete and odontocete cetaceans. Proc Roy Soc London B 270: 673-679.

2. Stamatakis A (2006) RAxML-VI-HPC: Maximum likelihood-based phylogenetic analyses with thousands of taxa and mixed models. Bioinformatics 22: 2688-2690.

3. Yang Z (2007) PAML 4: a program package for phylogenetic analysis by maximum likelihood. Mol Biol Evol 24: 1586-1591.

4. Meredith RW, Gatesy J, Murphy WJ, Ryder OA, Springer MS (2009) Molecular decay of the tooth gene enamelin (*ENAM*) mirrors the loss of enamel in the fossil record of placental mammals. PLoS Genet 5: e1000634.

5. Yang Z, Wong WSW, Nielsen R (2005) Bayes empirical Bayes inference of amino acid sites under positive selection. Mol Biol Evol 22: 1107-1118.

6. Zhang J, Nielsen R, Yang Z (2005) Evaluation of an improved branch-site likelihood method for detecting positive selection at the molecular level. Mol Biol Evol 22: 2472-2479.
